# Bioactive Compounds from Terrestrial and Marine-Derived Fungi of the Genus *Neosartorya* †

**DOI:** 10.3390/molecules27072351

**Published:** 2022-04-06

**Authors:** Joana D. M. de Sá, Decha Kumla, Tida Dethoup, Anake Kijjoa

**Affiliations:** 1 Laboratório de Química Orgânica, Departamento de Ciências Químicas, Faculdade de Farmácia, Universidade do Porto, Rua de Jorge Viterbo Ferreira, 228, 4050-313 Porto, Portugal; joanadmsa2703@gmail.com; 2 ICBAS—Instituto de Ciências Biomédicas Abel Salazar and CIIMAR, Rua de Jorge Viterbo Ferreira, 228, 4050-313 Porto, Portugal; decha1987@hotmail.com; 3 Department of Plant Pathology, Faculty of Agriculture, Kasetsart University, Bangkok 10240, Thailand; tdethoup@yahoo.com

**Keywords:** *Neosartorya*, Trichocomaceae, indole alkaloids, meroterpenoids, polyketides, anticancer, antibacterial activity

## Abstract

Fungi comprise the second most species-rich organism group after that of insects. Recent estimates hypothesized that the currently reported fungal species range from 3.5 to 5.1 million types worldwide. Fungi can grow in a wide range of habitats, from the desert to the depths of the sea. Most develop in terrestrial environments, but several species live only in aquatic habitats, and some live in symbiotic relationships with plants, animals, or other fungi. Fungi have been proved to be a rich source of biologically active natural products, some of which are clinically important drugs such as the β-lactam antibiotics, penicillin and cephalosporin, the immunosuppressant, cyclosporine, and the cholesterol-lowering drugs, compactin and lovastatin. Given the estimates of fungal biodiversity, it is easy to perceive that only a small fraction of fungi worldwide have ever been investigated regarding the production of biologically valuable compounds. Traditionally, fungi are classified primarily based on the structures associated with sexual reproduction. Thus, the genus *Neosartorya* (Family Trichocomaceae) is the telemorphic (sexual state) of the *Aspergillus* section known as *Fumigati*, which produces both a sexual state with ascospores and an asexual state with conidiospores, while the *Aspergillus* species produces only conidiospores. However, according to the Melbourne Code of nomenclature, only the genus name *Aspergillus* is to be used for both sexual and asexual states. Consequently, the genus name *Neosartorya* was no longer to be used after 1 January 2013. Nevertheless, the genus name *Neosartorya* is still used for the fungi that had already been taxonomically classified before the new rule was in force. Another aspect is that despite the small number of species (23 species) in the genus *Neosartorya,* and although less than half of them have been investigated chemically, the chemical diversity of this genus is impressive. Many chemical classes of compounds, some of which have unique scaffolds, such as indole alkaloids, peptides, meroterpenes, and polyketides, have been reported from its terrestrial, marine-derived, and endophytic species. Though the biological and pharmacological activities of a small fraction of the isolated metabolites have been investigated due to the available assay systems, they exhibited relevant biological and pharmacological activities, such as anticancer, antibacterial, antiplasmodial, lipid-lowering, and enzyme-inhibitory activities.

## 1. Introduction

The serendipitous discovery of penicillin by Alexander Fleming in 1928, as a bioactive principle from the culture broth of *Penicillium notatum* that inhibited the growth of Gram-positive bacteria, and its introduction in 1941 as an efficient antibacterial therapeutic without substantial side effects have been considered a hallmark of fungal bioactive compounds [1]. Furthermore, this extraordinary incident was followed by successive important events that strengthened the importance of terrestrial and marine-derived fungi as sources of useful bioactive compounds. Thus, in 1948, the Italian scientist Giuseppe Brotzu first observed the antibiotic properties of and then isolated a cephalosporin-producing fungus, *Cephalosporium acremonium* (which is known today as *Acremonium*), from a sewer on the Sardinian coast. However, it was only in 1962 that Dr. Abraham’s research team was able to produce cephalosporin C, the parent molecule of a new generation of antibiotics [2].

Fungi are also a source of cholesterol-lowering agents known as statins, which were blockbuster drugs in the 1990s. The story of statins started with the isolation of a hydroxymethyl glutaryl CoA reductase (HMGR) inhibitor, compactin (ML-236B), from the culture broth of a blue-green mold, *Penicillium citrinum* Pen-51, which was isolated from a rice sample by Akira Endo from Sankyo Research Laboratories in Tokyo. At the same time, Alfred Alberts and his colleagues at Merck Research Laboratories discovered a new natural product in a fermentation broth of *Aspergillus terreus*, which showed good HMGR inhibition; they named the product mevinolin, which later became known as lovastatin. Although Sankyo had discontinued the clinical development of compactin in 1980, its derivative, pravastatin, and lovastatin are natural statins of fungal origin [3]. Besides being important producers of valuable molecules in the field of drug discovery [4], fungi also play important roles in the environment and have the ability to exploit almost all niches, either natural or man-made. As such, some fungi are being used in the bioremediation of industrial waste [5,6]. Moreover, through industrial fermentation, they are also important components in industrial applications for the production of diverse ingredients (such as acidulants, enzymes, flavors, vitamins, colorants, and polyunsaturated fatty acids) used in food processing [7]. Fungi and fungal extracts are also being exploited in pest management programs to control pests and diseases [8].

Fungi are classified primarily based on the structures associated with sexual reproduction, which tend to be evolutionarily conserved. However, many fungi reproduce only asexually, and cannot easily be classified based on their sexual characteristics: some produce both asexual and sexual states. These problematic species are often members of the Ascomycota. Historically, Article 59 of the International Code of Botanical Nomenclature (Tokyo Code) permitted mycologists to give asexually reproducing fungi (anamorphs) separate names from their sexual states (teleomorphs). Thus, teleomorphic species belonging to the “*Aspergillus fischeri* series” of the *A. fumigatus* group (Raper and Fennell 1965) were placed in the genus *Neosartorya* (family Trichocomaceae) by Malloch and Cain (1972). While *Neosartorya* species produce both a sexual state with ascospores and an asexual state with conidiospores, the *Aspergillus* species produce only conidiospores [9]. The *Fumigati* include more than 20 *Neosartorya* species [10]. The dual naming system can be confusing and the separate names for the anamorphs of fungi with a pleomorphic life cycle have been an issue of debate since the phenomenon was recognized. In recent years, an increasing number of mycologists have recognized the urgent need for a transition to a single-name nomenclatural system for fungi, which resulted in the preparation of the “Amsterdam Declaration on Fungal Nomenclature”, under the auspices of the International Commission on the Taxonomy of Fungi (ICTF) during the symposium “One Fungus = One Name” held in Amsterdam in April 2011 [11]. The discontinuation of the dual nomenclature system was finally approved and adopted at the 18th International Botanical Congress in Melbourne in July 2011, during which the Vienna edition of the “International Code of Botanical Nomenclature” was replaced by the “International Code of Nomenclature for Algae, Fungi and Plants” (the Melbourne Code), published in December 2012. According to the Melbourne Code, one fungus can have only one name after 1 January 2013 [11,12]. Consequently, only the genus name *Aspergillus* was used for both sexual and asexual states after this rule was established. However, the genus name *Neosartorya* is still used for those fungi that had already been taxonomically classified before the new rule was in force.

Besides a small number of species (23 species) of the genus *Neosartorya* [9] when compared to the genus *Aspergillus,* which comprises a large number of species (339 species) [13], only half of them (*N. fischeri*, *N. pseudofischeri*, *N. glabra*, *N. tsunodae*, *N. laciniosa*, *N. paulistensis*, *N. fenelliae*, *N. spinosa*, *N. quadricincta*, *N. takakii*, *N. hiratsukae*, *N. udagawae* and *N. siamensis*) have been investigated for their secondary metabolites. Despite this, we have found great chemical diversity and numerous interesting biological/pharmacological activities in secondary metabolites produced by members of the genus *Neosartorya*. Although our group has previously reviewed the bioactive secondary metabolites from a Thai collection of soil and marine-derived fungi of the genera *Neosartorya* and *Aspergillus* [14], this review reports 213 secondary metabolites isolated from cultures of terrestrial, marine-derived and endophytic fungi of the members of the *Neosartorya* genus, covering the literature published from 1993 to 2021. The relevant biological and pharmacological activities of some metabolites are also highlighted when applicable. The search engines that were used to find the reports of compounds included in this review were PubMed, MEDLINE, Web of Science, and Scopus.

## 2. Secondary Metabolites Produced by the Cultures of Fungi of the Genus *Neosartorya*

Since *Neosartorya* is a teleomorph of the *A. fumigatus* group, it is a legitimate expectation that, in principle, secondary metabolites produced by members of the genus *Neosartorya* would resemble those produced by *Aspergillus* species. In fact, we have found common traits in the secondary metabolites produced by *Neosartorya* species and *Aspergillus* species, especially indoles [15], meroterpenoids [16], and polyketides [17]. Surprisingly, we have noted different types of metabolites within the same species, isolated from different environments. However, it is not possible to conclude whether the fungus isolated from different environments produced different metabolites or whether the culture media used also plays a role in this phenomenon.

In the following subsections, the secondary metabolites are categorized according to their biosynthetic origins and are then subdivided according to their structural features.

### 2.1. Indole Alkaloids

Indole alkaloids consist of the indole ring system, which is derived from the amino acid Trp. In order to facilitate the readability of this section, these compounds are subdivided into simple indoles, prenylated indoles, annelated indoles, and bis-indoles, according to Wibowo et al. [15].

#### 2.1.1. Simple Indoles

**Figure 1:**
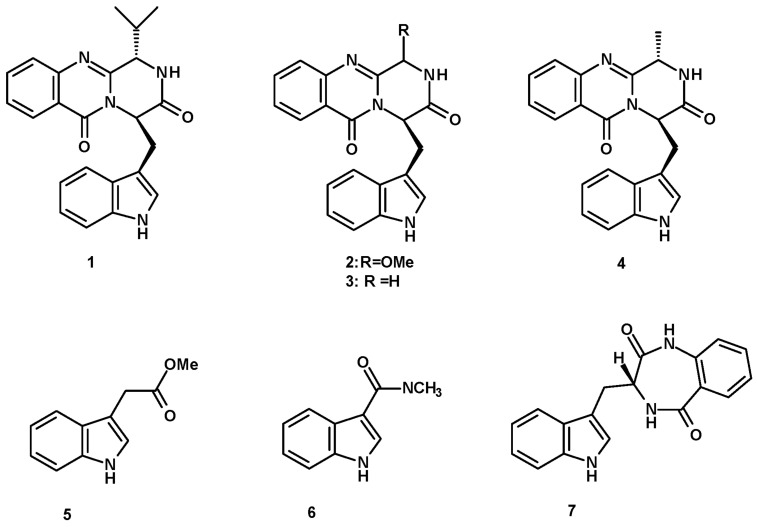
Structures of simple indoles **1**–**7**.

Fiscalin B (**1**) (Figure 1), a simple indole having an isopropyl pyrazinoquinazolinone ring system, linked to an indole moiety by a methylene group, was first isolated in 1993 from the culture extract of *N. fischeri*, obtained from a plant rhizosphere that was collected near the We Fung Chi Cascade region of Taiwan. The fungus was cultured in a liquid medium containing glycerol, dextrin, Bacto-Soytone, yeast extract, (NH_4_)_2_SO_4_, and CaCO_3_ at pH 7.0 [18]. Later, this compound was also obtained from the extract of marine-derived *N. fischeri*, isolated from a marine mud collected in the intertidal zone of Hainan province, China, and cultured in a liquid medium (barley sugar, ajinomoto, glucose, yeast extract, steepwater, mannitol, KH_2_PO_4_, MgSO_4_·7H_2_O, CaCO_3_, salt, with a pH of 6.5) [19].

Two pyrazinoquinazolinone-containing indoles, i.e., the unreported 3-methoxyglyantrypine (**2**) and the previously described glyantrypine (**3**) (Figure 1) were also obtained from a culture (cooked rice) extract of *N. fischeri* TJ 403-CA8, isolated from a medicinal insect, *Cryptotympana atrata* (Cicadidae), which was collected from the Pangquan Ditch National Nature Reserve of Lvliang City, Shanxi Province, China [20]. Fumiquinazoline F (**4**), a methyl pyrazinoquinazolinone-containing indole, and indolyl-3-acetic acid methyl ester (**5**) (Figure 1) were isolated from a liquid culture extract (glucose, peptone, yeast extract, CaCO_3_, H_2_O) of the marine-derived *N. pseudofischeri*, isolated from the inner tissue of a starfish (*Acanthaster planci*) that was collected from the Hainan Sanya Natural Coral Reef Reserve, China [21].

The extract of marine-derived *N. pseudofischeri* (collection no. 2014F27-1), isolated from the inner tissue of a sea star (*A. planci*), was collected from Hainan Sanya National Coral Reef Reserve, China, and cultured in glucose-peptone-yeast extract medium, furnishing *N*-methyl-1*H*-indole-2-carboxamide (**6**) (Figure 1) [22].

In addition, (3*R*)-3-(1*H*-indol-3-ylmethyl)-3, 4-dihydro-1*H*-1,4-benzodiazepine-2,5-dione (**7**) (Figure 1) was obtained from a culture extract of the marine sponge-associated fungus *N. glabra* KUFA 0702, isolated from the marine sponge *Mycale* sp., which was collected from the coral reef at Samaesarn Island in the Gulf of Thailand, Chonburi Province, and cultured in a cooked rice solid medium [23].

#### 2.1.2. Prenylated Indoles

##### 1,4-Benzodiazepene-2,5-dione-containing Prenylated Indoles

**Figure 2:**
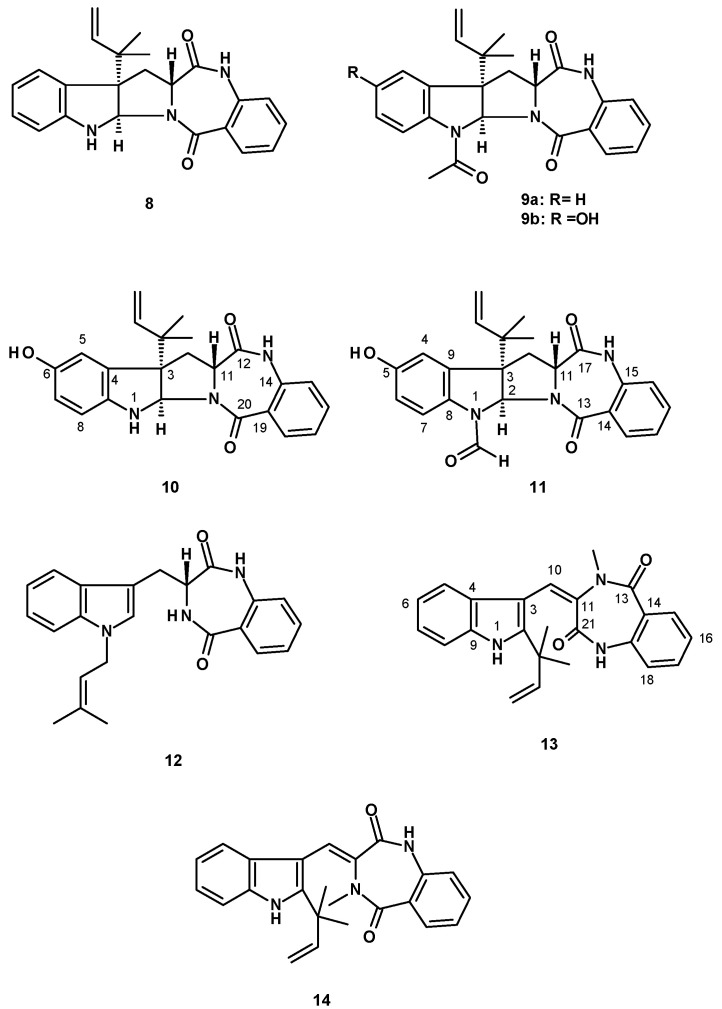
Structures of prenylated indoles **8**–**14**.

Aszonalenin (**8**) and acetylaszonalenin (**9a**) (Figure 2) are the most frequently isolated 1,4-benzodiazepene-2,5-dione-containing prenylated indoles from the genus *Neosartorya.* Compound **8** was isolated from a culture extract of: *N. pseudofischeri* IFM 52672, which was cultured in moist rice [24]; *N. ficheri* KUFC6344, isolated from coastal forest soil at Samaesarn island, Chonburi Province, Thailand, and cultured in a cooked rice solid medium [25]; *N. fischeri* CGMCC3.5378, obtained from the Chinese Academy of Science, and cultured in a solid medium containing moist corn germ [26]; *N. fischeri* JS0553, an endophytic fungus isolated from the plant *Glehnia littoralis* (family Apiaceae), which was collected in a swamp area of Suncheon, South Korea, and cultured in a solid rice medium [27]; *N. tatenoi* KKU-2NK23, collected from forest soil in Khon Kaen province, Thailand, and cultured in a liquid medium containing potato dextrose broth [28]; the marine-derived *N. takakii* KUFC 7898, isolated from the alga *Amphiroa* sp., which was collected in Samaesarn Island in the Gulf of Thailand and cultured in a cooked rice solid medium [29], the marine-derived *N. glabra*, isolated from a marine sponge *Mycale* sp., which was collected from the coral reef of Samaesarn Island, in the Gulf of Thailand, and cultured in a cooked rice solid medium [23]; and the marine sponge-associated *N. fenelliae* KUFA 0811, isolated from the marine sponge *Clathria reinwardtii,* which was collected from Samaesarn Island in the Gulf of Thailand and cultured in a cooked rice solid medium [30]. Compound **9a** was reported only from *N. ficheri* KUFC6344 [25], *N. fischeri* CGMCC3.5378 [26], an endophytic fungus, *N. fischeri* JS0553 [27], and *N. fischeri* TJ 403-CA8 [20], whereas the unreported 6-hydroxyacetylaszonalenin (**9b**) (Figure 2) was also isolated from a culture extract of the insect-derived *N. fischeri* TJ403-CA8 [20]. The indole 6-Hydroxyaszonalenin (**10**) (Figure 2) was also isolated from a culture extract of *N. fischeri* CGMCC3.5378 [26] and the insect-derived *N. fischeri* TJ 403-CA8 [20], whereas 1-formyl-5-hydroxyaszonalenin (**11**) (Figure 2) was obtained from a culture extract of *N. fischeri* KUFC 6344 [25]. 

Tetracyclic 1,4-benzodiazepene-2,5-dione-containing prenylated indoles were also reported from the fungi of the genus *Neosartorya*. Whereas takakiamide (**12**) (Figure 2) was isolated from a culture extract of the marine-derived *N. takakii* KUFC 7898 [29] and later from a culture extract of the marine-derived *N. glabra* [23], fischeramides A (**13**) and B (**14**) (Figure 2) were also isolated from the insect-derived *N. fischeri* TJ 403-CA8 [20].

##### 1,4-Diketopiperazine-containing Prenylated Indoles 

The indole 1,4-diketopiperazine-containing prenylated indoles are a large group of indole alkaloids reported from members of the genus *Neosartorya*. Most of the isolated compounds are pentacyclic, but tetra- or hexacyclic compounds were also reported. They can be mono-, di- or triprenylated. Normally, the 1,4-diketopiperazine moiety is linearly fused with a pyrrolidine ring to form a hexahydropyrrolo [1,2-*a*]pyrazine-1,4-dione ring system, evidencing the incorporation of the amino acid proline in their biogenesis.

**Figure 3:**
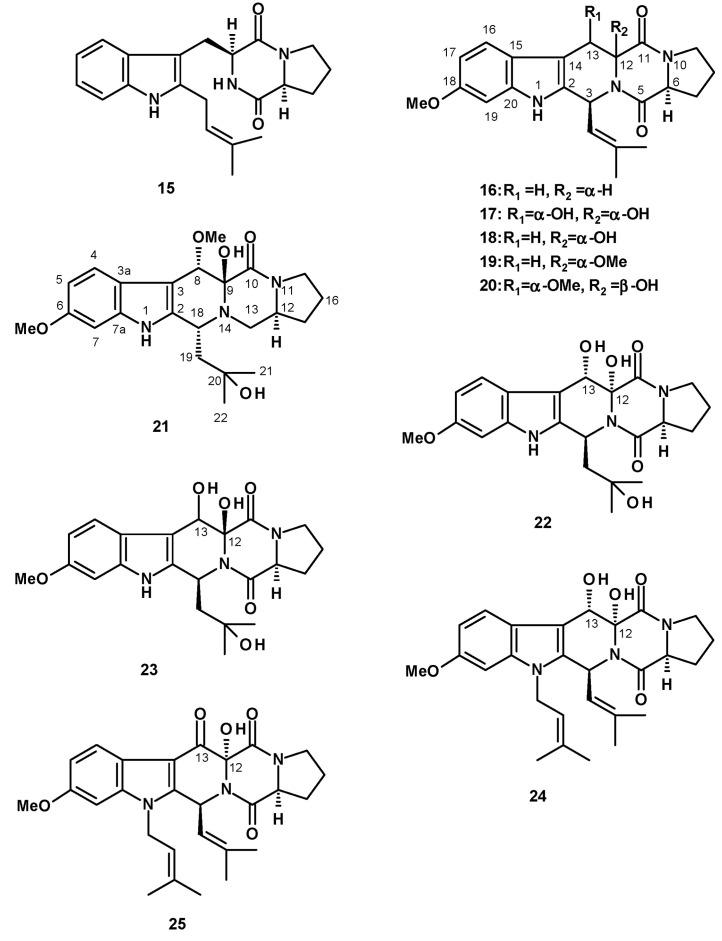
Structures of prenylated indoles **15**–**25**.

Tryprostatin B (**15**) (Figure 3), a tetracyclic 1,4-diketopiperazine-containing prenylated indole, was isolated from the insect-derived *N. fischeri* TJ 403-CA8 [20], while the pentacyclic analog, fumitremorgin C (**16**) (Figure 3) was reported as being sourced from culture extracts of *N. fischeri* CGMCC 3.5378 (from the Chinese Academy of Science), which were cultured in a wheat bran solid medium [31,32]. Analogs of fumitremorgin C (**16**), i.e., 12α,13α-dihydroxyfumitremorgin C (**17**), 12-hydroxyfumitremorgin C (**18**), 12-methoxyfumitremorgin C (**19**), cyclotrypostatin B (**20**), *rel*-(8*S*)-19,20-dihydro-8-methoxy-9,18-diepifumitremorgin C (**21**) and verruculagen TR-2 (**22**) (Figure 3) were also reported from *N. fischeri* CGMCC 3.5378 [32]. Compounds **18**–**20** were also isolated from the insect-derived *N. fischeri* TJ403-CA8 [20], while 12β-hydroxyverruculagen TR-2 (**23**) (Figure 3) was isolated from a culture extract of *N. fischeri* NRRL 181, purchased from DSMZ (DE-Braunschweig), and was cultured in potato dextrose agar medium [33]. Compound **20** was also isolated, together with fumitremorgin B (**24**) (Figure 3), from the endophytic fungus, *N. fischeri* JS0553 [27]. Compound **24** was also isolated from *N. fischeri* var. *fischeri* CBM-FA-0156, which was cultured in a solid rice medium [34], whereas 13-oxofumitremorgin B (**25**) (Figure 3) was isolated from a culture extract of the soil fungus *N. fischeri* KUFC 6344 [25], as well as from *N. fischeri* CGMCC 3.5378 [26].

**Figure 4:**
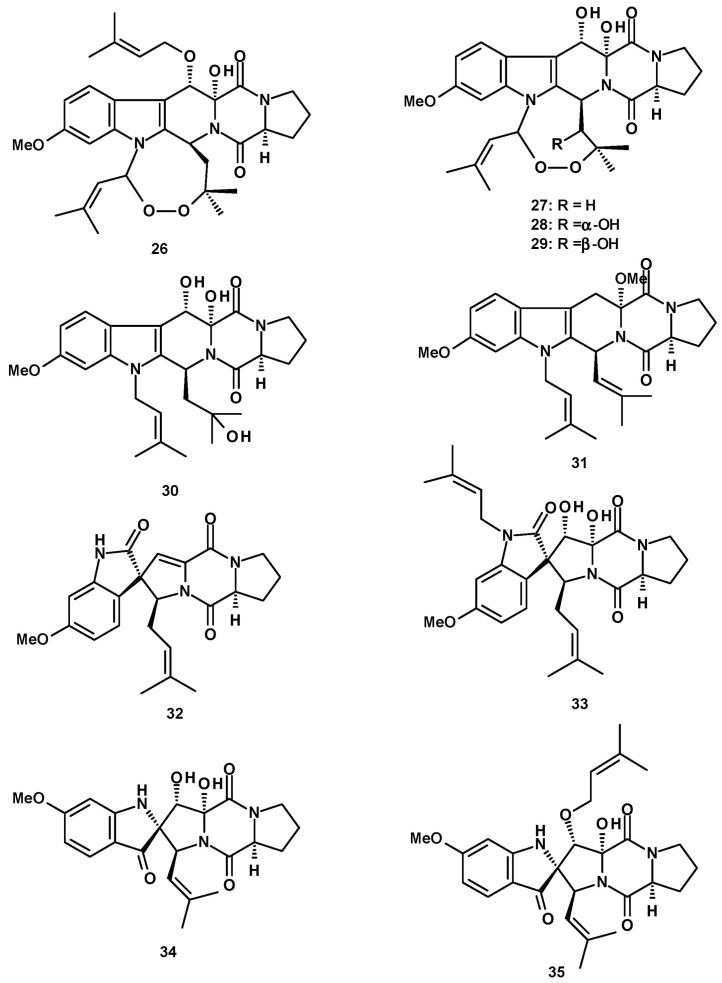
Structures of prenylated indoles **26**–**35**.

Fumitremorgin A (**26**) (Figure 4), a hexacyclic 1,4-diketopiperazine-containing prenylated indole with a peroxide-containing eight-membered ring formed by two prenyl groups, was reported from the endophytic fungus *N. fischeri* JS0553 [27], *N. fischeri* NRRL 18 [33], and *N. fischeri* var. *fischeri* CBM-FA-0156 [34].

Three peroxide-containing hexacyclic prenylated indoles, including the previously reported verruculogen (**27**) and two undescribed neofipiperazines A (**28**) and B (**29**), together with an undescribed pentacyclic diprenylated indole, neofipiperazine C (**30**) (Figure 4) were isolated from a culture extract of *N. fischeri* CGMCC 3.5378 [32]. Compound **27** was also reported from *N. fischeri* CGMCC 3.5378 [26] and *N. fischeri* NRRL 181 [33]. Neofipiperazine D (**31**) (Figure 4), another undescribed pentacyclic diprenylated indole, was isolated from a culture extract of *N. fischeri* CGMCC 3.5378 [31].

Previously reported 6-methoxyspirotryprostatin B (**32**), spirotryprostatin C (**33**), spiro [5*H*,10*H*-dipyrrolo [1,2-*a*]:1′,2′-*d*]pyrazine-2-[3*H*], 2′[2*H*]indole]-3,5,10(1′*H*]trione (**34**), and the unreported spirotryprostatin M (**35**) (Figure 4) were isolated from a culture extract of the insect-derived *N. fischeri* TJ 403-CA8 [20].

##### Quinazolinone-Containing Prenylated Indoles

**Figure 5:**
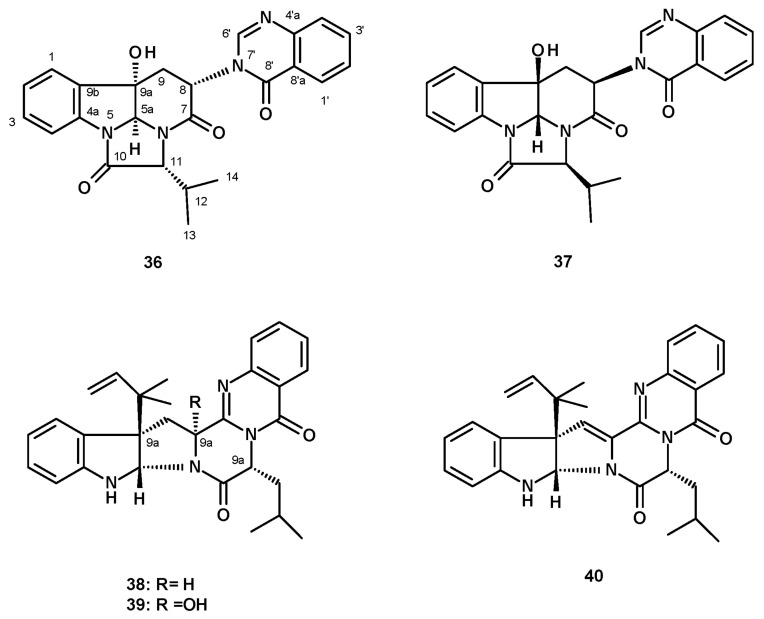
Structures of prenylated indoles **36**–**40**.

Pseudofischerine (**36**) (Figure 5), an unreported quinazolinone-containing prenylated indole, was isolated from a culture extract of *N. pseudofischeri* KUFC 6422 S. W. Peterson, obtained from soil planted with rose apples (*Eugenia javanica*, family Myrtaceae) from Angthong Province, Thailand, and cultured in a cooked rice solid medium [35]. The structure of the compound was established by the interpretation of high-resolution mass spectrum (HRMS) and 1-dimensional (1D) and 2-dimensional (2D) NMR data. The relative stereochemistry of **36** was established, based on the NOESY correlations from H-5a to OH-9a, H-12, Me-13, Me-14, H-6′, as well as via comparison with the structure of the previously described chaetominine, isolated from the endophytic fungus *Chaetomium* sp. IFB-E015, the stereochemistry of which was established by single-crystal X-ray analysis and the determination of the aminoacid L-Ala using Marfey’s method [36]. Later on, Liao et al. reported the isolation of isochaetominine C (**37**) from a culture extract of the marine-derived *Aspergillus* sp. (strain number F452). Surprisingly, the ^1^H and ^13^C NMR data of **37** and **36** (both in DMSO-*d*_6_) were nearly identical; however, the stereochemistry of **37** was enantiomeric of **36**. Since the configurations of C-5a, C-8, C-9a and C-11 in **37** were determined by NOESY correlations and an identification of the amino acid L-Val using an advanced Marfey’s method [37], the absolute configurations of its stereogenic carbons were established. Therefore, **36** and **37** are the same compound. Later on, Lan et al. [21] reported the isolation of isochaetominine C (**37**) from a culture extract of *N. pseudofischeri*, isolated from the inner tissue of a starfish (*A. planci*) that was collected from the Hainan Sanya National Coral Reef Reserve, China, and cultured in a liquid medium. However, the stereochemistry of the structure of isochaetominine C reported in this paper is opposite to that reported by Liao et al. [37]. Compound **37** was also isolated from *N. pseudofischeri* [38] and also from *N. hiratsukae* [39]; both samples were collected from soil in the Chiang Mai forest, Thailand, and cultured in a potato dextrose liquid medium.

Three previously unreported reverse prenylated indole alkaloids analogs of (-)-ardeemins, sartoryglabrins A (**38**), B (**39**) and C (**40**) (Figure 5), were isolated from an extract of a solid culture medium (cooked rice) of *N. pseudofischeri*, which was collected from soil in Chonburi Province, Thailand. The structures of the compounds were elucidated by analysis of HRMS, 1D and 2D NMR data. The absolute structure of **38** was established by X-ray analysis, using CuKα radiation [40].

#### 2.1.3. Anellated Indoles

Like prenylated indoles, anellated indoles also constitute a large group of specialized metabolites reported from both terrestrial and marine-derived *Neosartorya* species. Their structures vary from simple to complex, and some of them incorporate sulfur atoms to form a disulfide bridge.

##### β-Carboline Alkaloids

**Figure 6:**
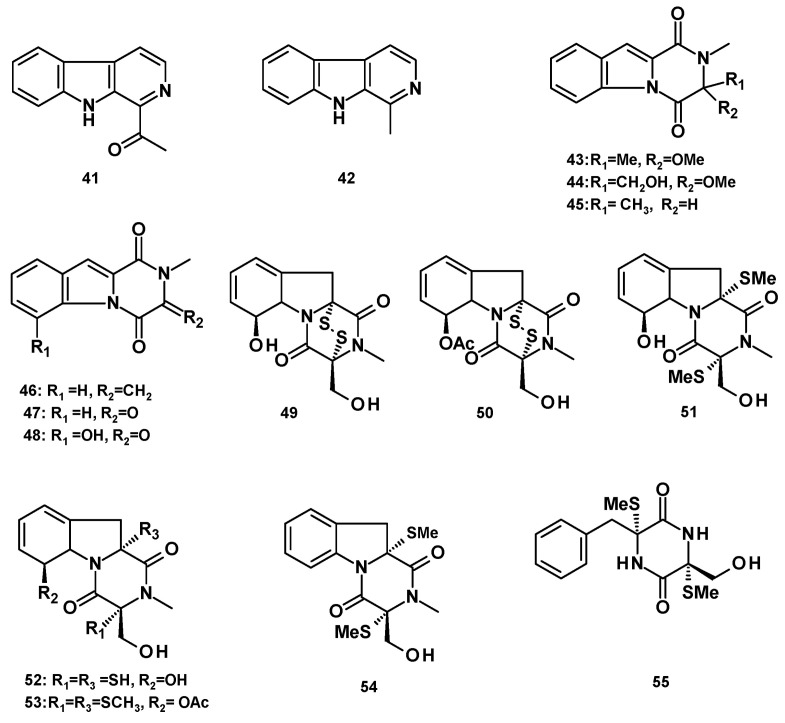
Structures of anellated indoles **41**–**55**.

Two β-carboline analogs, 1-acetyl β-carboline (**41**) [21] and harmane (**42**) [30] (Figure 6) were isolated from a culture extract of the marine-derived *N. pseudofischeri*, isolated from the inner tissue of a starfish (*A. planci*) and the marine sponge-associated *N. tsunodae* KUFC 9213, respectively.

##### Pyrazino [1,2-a]indole-1,4-dione Derivatives

Two unreported 2,3-dihydropyrazino [1,2-*a*]indole-1,4-dione derivatives, neosartins A (**43**) and B (**44**), and the previously reported 1,2,3,4-tetrahydro-2-methyl-1,4-dioxopyrazino [1,2-*a*]indole (**45**), 1,2,3,4-tetrahydro-2-methyl-3-methylene-1,4-dioxopyrazino [1,2-*a*]indole (**46**), 1,2,3,4-tetrahydro-2-methyl-1,3,4-trioxopyrazino [1,2-*a*]indole (**47)**, were isolated from a culture extract of the marine-derived *N. pseudofischeri* (collection no. 2014F27-1) [22], while the previously reported 6-hydroxy analog of **47**, 1,2,3,4-tetrahydro-6-hydroxy-2-methyl-1,3,4-trioxopyrazino [1,2-*a*]indole (**48**), was isolated from a culture extract of the marine-derived *N. pseudofischeri*, isolated from the inner tissue of a starfish (*A. planci*) [21].

The sulfur-containing hexahydropyrazino [1,2-*a*]indole-1,4-dione analogs, gliotoxin (**49**), acetylgliotoxin (**50**), bis(dethio)bis(methylthio)gliotoxin (**51**), reduced gliotoxin (**52**), 6-acetylbis(methylthio)gliotoxin (**53**), didehydrobisdethiobis (methylthio)gliotoxin (**54**) and bis-*N*-norgliovictin (**55**) (Figure 6) were also reported from a culture extract of the marine-derived *N. pseudofischeri* (collection no. 2014F27-1) [22]. Compounds **49** and **51** were also reported from a culture extract of *N. pseudofischeri*, cultured in a solid rice medium [41].

##### Quinazolinone-Containing Anellated Indoles

**Figure 7:**
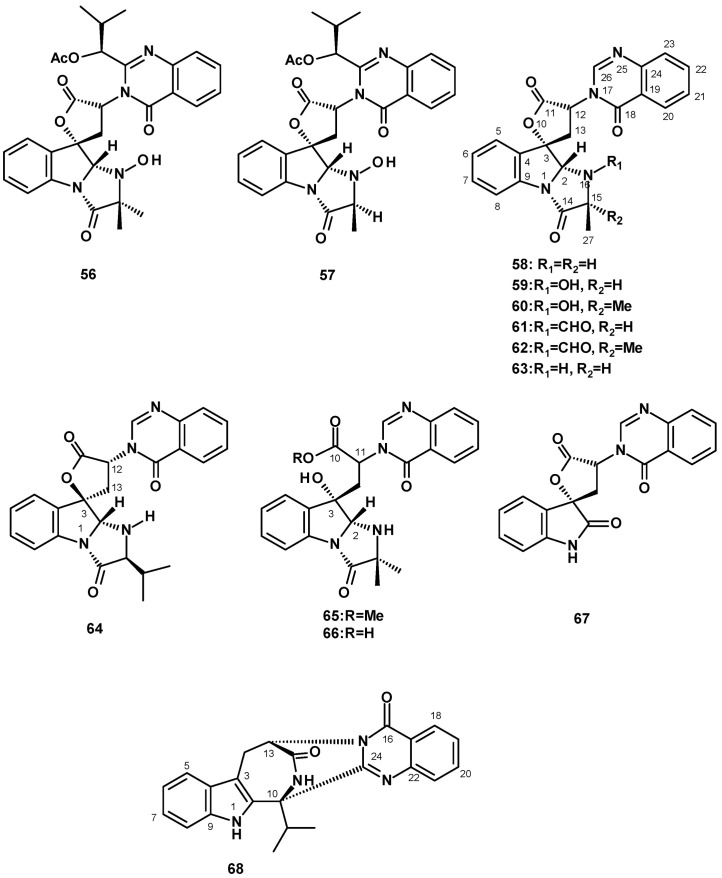
Structures of anellated indoles **56**–**68**.

A major group of quinazolinone-containing anellated indoles are the tryptoquivalines. The structural characteristic of tryptoquivalines is the presence of a quinazolinone moiety, connected to the *6-5-5*-imidazoindolone ring system via a five-membered spirolactone. Tryptoquivaline (**56**) (Figure 7) was isolated from a solid culture (cooked rice) extract of *N. siamensis* KUFC 6349 obtained from forest soil on Samaesarn Island, Chonburi province in Thailand [42], the marine-derived *N. siamensis* KUFA 0017, isolated from a sea fan (*Rumphella* sp.) that was collected from the coral reef of the Similan Islands, Thailand [43], and *N. spinosa* KKU-1NK1, obtained from forest soil in Khon Kaen Province, Thailand [44], whereas nortryptoquivaline (**57**) (Figure 7) was isolated from a culture extract of *N. pseudofischeri* IFM 52672 [24], the marine-derived *N. siamensis* KUFCA0017 [43], and also from a soil-derived *N. spinosa* KKU-1NK1 [44].

The previously reported tryptoquivalines F (**58**), H (**59**), L (**60**), and the unreported tryptoquivaline O (**61**) (Figure 7) were also isolated from a culture extract of the soil-derived *N. siamensis* KUFC 6349. It is worth mentioning that Buttachon et al. [42] have established the absolute configurations of C-2, C-3, and C-12 of **60** as 2*S*, 3*S*, 12*R* by X-ray analysis using CuKα radiation, which was opposite to those previously reported by Yamazaki et al. [45], thus establishing unambiguously the stereostructures of the tryptoquivaline series. Another unreported tryptoquivaline analog, tryptoquivaline T (**62**), was isolated from a culture extract of the diseased coral-derived *N. laciniosa* KUFC 7896 [46]. Compounds **58**–**60** were also isolated from a culture extract of the marine sponge-associated *N. paulistensis* KUFC 7897 [46]. Compound **60** was the most common tryptoquivaline, being reported from various species and strains of *Neosartorya*, such as the marine-derived *N. siamensis* KUFCA0017 [43], the marine-derived *N. laciniosa* KUFC 7896 [25], and the soil-derived *N. spinosa* KKU-1NK1 [44]. Compound **59** was also reported from the marine-derived *N. siamensis* KUFA 0017 [43], the marine sponge-associated *N. paulistensis* KUFC 7897 [46]. Compounds **59** and **60** were isolated, together with a new tryptoquivaline analog, tryproquivaline U (**63**), from a culture extract of the algicolous fungus, *N. takakii* KUFC 7898 [29].

The unreported tryptoquivaline V (**64**) was isolated from a culture extract of the soil-derived *N. pseudofischeri* [38]. It is interesting to note that the stereochemistry at C-3 of the five-membered lactone was opposite to that of all the reported tryptoquivalines. The authors determined the absolute configuration of C-3 only by NOESY correlations between key protons, some of which were not well defined, and a sign of the optical rotation. However, the authors did not use any reliable methods, such as X-ray crystallography with CuKα radiation or chiroptical methods, to determine the absolute configuration of the stereogenic carbon.

Two tryptoquivaline derivatives, the tryptoquivalines P (**65**) and Q (**66**) (Figure 7), were isolated from the organic extract of *Neosartorya* sp. HN-M-3, obtained from a marine mud in the intertidal zone of Hainan Province, China, and cultured in a liquid medium containing barley sugar, ajinomoto, glucose, and yeast extract. The structures of **65** and **66** differ from other tryptoquivalines in that the five-membered lactone ring is hydrolyzed to give a hydroxy group on C-2 and a carboxylic acid on C-11. However, the absolute configurations at C-2 and C-3 were not determined [47].

The indole 3′-(4-Oxoquinazolin-3-yl)spiro [1*H*]-indole-3,5′]-2,2′-dione (**67**) (Figure 7), which contains a quinazolinone moiety connected to 2-oxindole instead of the *6-5-5*-imidazoindolone ring system, via a five-membered spirolactone, was first isolated from a culture extract of *N. siamensis* KUFC 6349 [42] and, later, from the sea-fan-derived *N. siamensis* KUFA 0017 [43], the marine-derived *N. laciniosa* KUFC 7896 [25], the marine-derived *N. paulistensis* KUFC 7897 [46], and the marine-derived *N. takakii* KUFC7898 [29].

The undescribed quinazolinone-containing hexacyclic indole alkaloid consisting of an azepinone ring fused with the indole ring system, named sartorymensin (**68**) (Figure 7), was isolated from a culture extract of the soil-derived *N. siamensis* KUFC 6349. The structure of **68** was established by the interpretation of HRMS and 1D and 2D NMR data. The absolute configurations at C-10 and C-13 were established unequivocally as 10*S* and 13*S* by X-ray analysis using CuKα radiation [42].

##### Pyrazinoquinazolinone-Containing Anellated Indoles

**Figure 8:**
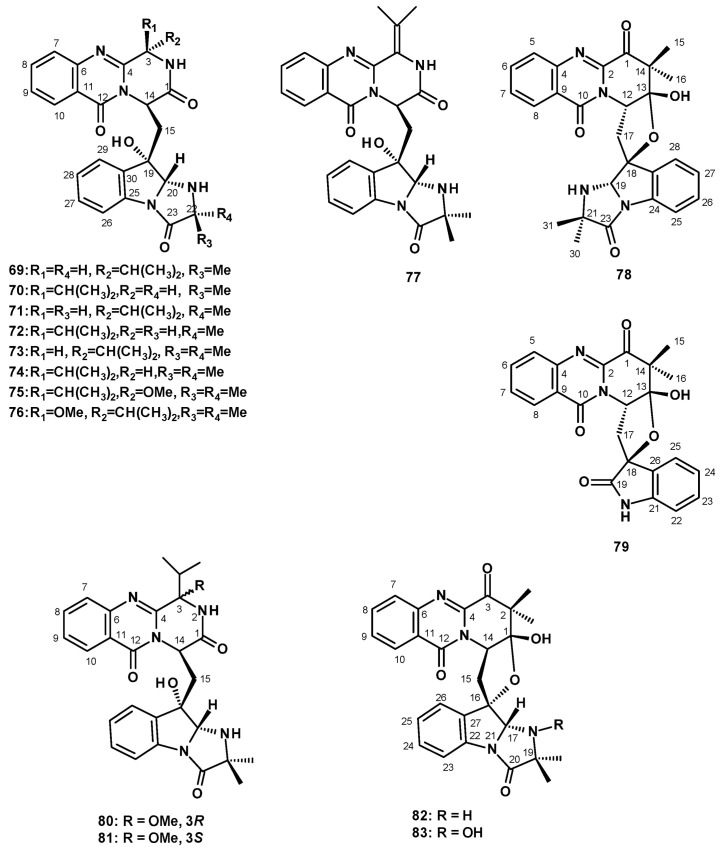
Structures of anellated indoles **69**–**83**.

The compounds of this group consist of a pyrazinoquinazolinone moiety connected to the *6-5-5*-imidazoindolone ring system by a methylene bridge. A culture extract of the soil-derived *N. siamensis* KUFC 6349 furnished the previously reported fiscalin A (**69**) and its undescribed diastereomers, *epi*-fiscalin A (**70**), neofiscalin A (**71**), and *epi*-neofiscalin A (**72**), as well as the previously reported fiscalin C (**73**) and the undescribed *epi*-fiscalin C (**74**) (Figure 8). The structures of **69**–**74** were elucidated by extensive analysis of HRMS data and 1D and 2D NMR spectral analysis. The configuration of C-3 in **70** was evidenced by a W-type long-range coupling between NH-2 and H-14 in the COSY spectrum, while the configurations of C-20 and C-22 were proved to be the same as those of **69** by a NOESY correlation from H-20 to Me-21. The stereostructures of **71** and **72** were based on a W-type long-range coupling between NH-2 and H-14 in the COSY spectrum and the NOESY correlation from H-20 to Me-21 or H-22. The structures and configurations of the stereogenic carbons of **69**–**72** were corroborated by the stereostructure of **73** and **74,** whose structures and the absolute configurations of the stereogenic carbons were established conclusively by X-ray analysis using CuKα radiation [42]. Compounds **69**–**74** were also isolated from a culture extract of the sea-fan-derived *N. siamensis* KUFA 0017 [43].

Compound **74** was also isolated, together with two unreported tryptoquivalines, E (**75**) and F (**76**) (Figure 8), from a culture extract of *N. udagawae* HDN 13-313 that was obtained from the root of a mangrove plant, *Aricennia marina*, collected from a mangrove conservation area in Hainan Province, China, and cultured in a liquid medium (composed of maltose, mannitol, glucose, monosodium glutamate, and yeast extract). The stereochemistry of **75** and **76** was established by a comparison of the calculated and experimental electronic circular dichroism (ECD) spectra. The structure and stereochemistry of **75** were also confirmed by X-ray analysis [48]. The previously reported fiscalin analog, quinadoline A (**77**) (Figure 8), was also isolated from the soil-derived *N. spinosa* KKU-1NK1 [44].

##### Pyridoquinazolinone-Containing Anellated Indoles

Wu et al. reported the isolation of an undescribed pyridoquinazolinone linked to 2-oxindole by a spirofuran ring, together with an undescribed pyridoquinazolinone linked to the imidazolindolone moiety by a spirofuran ring, which they have named tryptoquivalines U (**78**) and T (**79**) (Figure 8), respectively. These were from a culture extract of the marine-derived *N. fischeri*, isolated from a marine mud, which was collected in the intertidal zone of Hainan Province, China [19]. Interestingly, the authors were unaware of the existence of the previously reported tryptoquivaline T (**62)**, isolated from a culture extract of the diseased coral-derived *N. laciniosa* KUFC 7896 [46], along with tryptoquivaline U (**63)**, isolated from a culture extract of the algicolous fungus *N. takakii* KUFC 7898 [29], and they gave the same names to their compounds. Structurally, tryptoquivalines are a class of indole alkaloids, having a quinazolinone moiety connected to the *6-5-5*-imidazoindolone ring system via a five-membered spirolactone and not a pyridoquinazolinone connected to the *6-5-5*-imidazoindolone ring system via a five-membered spirolactone, which is the case with **78** and **79**.

Later, Yu et al. reported the undescribed fiscalins E **(80**) and F (**81**), and two pyridoquinazolinones, linked to the imidazolindolone ring system by a spirofuran ring, which were named *Neosartorya*dins A (**82**) and B (**83**) (Figure 8), and were taken from a culture extract of *N. udagawae* HDN 13-313 [48]. The structures of both compounds were established by extensive analysis of HRMS and 1D and 2D NMR data. The absolute configurations of the stereogenic carbons in **80** and **81** were established by comparison of calculated and experimental ECD spectra. In the case of **80**, the absolute structure was confirmed by X-ray analysis. The relative configurations of the stereogenic carbons in **82** and **83** were established by NOESY correlations of the key protons while the absolute configurations at C-1, C-14, C-16 and C-17 in **82** were determined as 1*R*, 14*R*, 16*S*, and 17*R* by comparison of the calculated and experimental ECD spectra. The absolute configurations at C-1, C-14, C-16 and C-17 in **83** are the same as those of **77,** since both compounds displayed nearly identical ECD spectra. Interestingly, the structure of *Neosartorya*din A (**82**) is the same as that of tryptoquivaline U (**78**), as reported by Wu et al. [19]. The only difference is that the configuration of C-12 in **78** is opposite to that of the same carbon (C-14) in **82**. Since the configuration of C-12 in **78** was opposite to that of the same carbon of all other imidazolindolone-containing compounds isolated from members of this genus, this raises the possibility of a wrong assignment.

**Figure 9:**
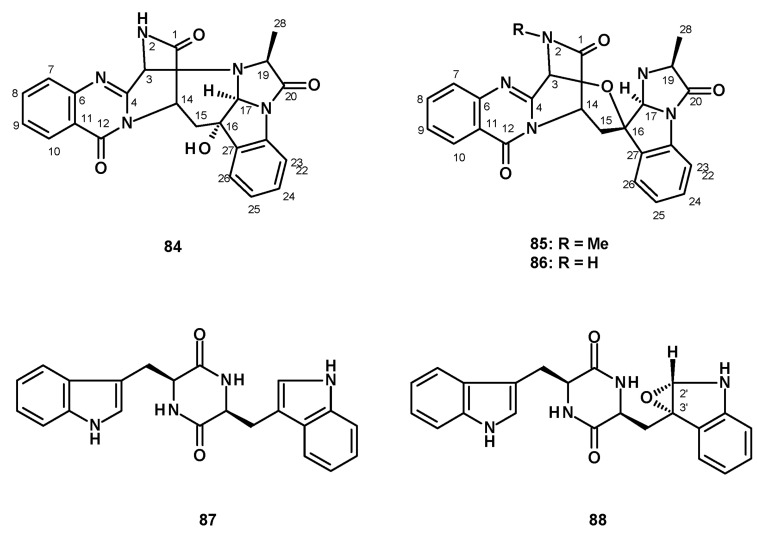
Structures of indoles **84**–**88**.

Shan et al. described the isolation of two undescribed norfumiquinazolines, cottoquinazolines E and F, from the ethanol extract of a solid culture (moist wheat) of *N. fischeri* NRRL 181 [49]. The structures of the compounds were elucidated by extensive analysis of 1D and 2D NMR and HRMS spectral data; however, the relative configurations of some stereogenic carbons were still undetermined by NOESY correlations. Recently, Lin et al. also obtained the cottoquinazolines E (**84**), F (**85**), and G (**86**) (Figure 9) from the organic extract of a solid rice culture of the insect-derived *N. fischeri* TJ 403-CA8. The structures of the compounds were established by analysis of HRMS and 1D and 2D NMR spectral data. The relative configurations of C-16, C-17 and C-19 were determined as 16S*, 17S*, and 19S* by NOESY correlations, while the absolute configurations of C-3, C-14, C-16, C-17, and C-19 were determined by X-ray analysis using CuKα radiation as 3*S*, 14*S*, 16*S*, 17*S*, and 19*S*, thus solving the structure and the absolute configurations of the stereogenic carbons in **84**. The absolute configurations of the stereogenic carbons in **85** and **86** were determined by comparison of their calculated and experimental ECD spectra [50].

#### 2.1.4. Bis-Indoles

Only three bis-indoles were isolated from fungi of the genus *Neosartorya*. Fellutamine A (**87**) and the unreported fellutamine A epoxide (**88**) (Figure 9) were isolated from a culture extract of the marine sponge-associated *N. glabra* KUFA 0702 [23]. The relative configurations of C-2′ and C-3′ in **88** were established by NOESY correlations, as well as by molecular modeling. Compound **87** was also isolated from the marine sponge-associated *N. fenelliae* KU0811 [30].

### 2.2. Dibenzylpiperazine Alkaloids

Although indole alkaloids are very copious in the fungi of the genus *Neosartorya*, dibenzylpiperazine alkaloids are very rare among the species investigated. Biosynthetically, dibenzylpiperazine alkaloids are derived from the coupling of Phe/Tyr.

**Figure 10:**
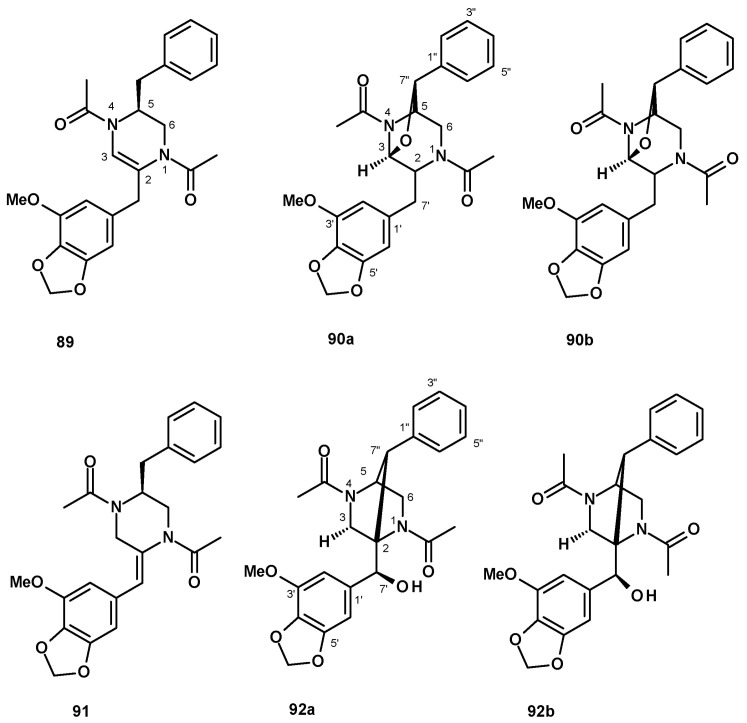
Structures of alkaloids **89**–**92**.

Eamvijarn et al. [35] described the isolation of an enantiomeric rotamer (**89**) (Figure 10) of the previously reported brasiliamide B [51] via analysis of the chemical shift values of H-3, H-5, and the methyl groups of the N_4_-acetamide, in addition to two rotamers of an undescribed 1,4-diacetyl-2,5-dibenzylpiperazine-3,7”-oxide (**90a**/**90b**) (Figure 10) from a culture extract of the soil-derived *N. pseudofischeri*. Compounds **90a**/**90b** were later isolated, together with the unreported brasiliamide G (**91**) (Figure 10), from a culture extract of the soil-derived *N. fischeri* [38], while both rotamers of the undescribed brasiliamide H (**92a**/**92b**) (Figure 10) were isolated from a culture extract of the soil-derived *N. hiratsukae* (specimen EU06) [39].

### 2.3. Peptides

**Figure 11:**
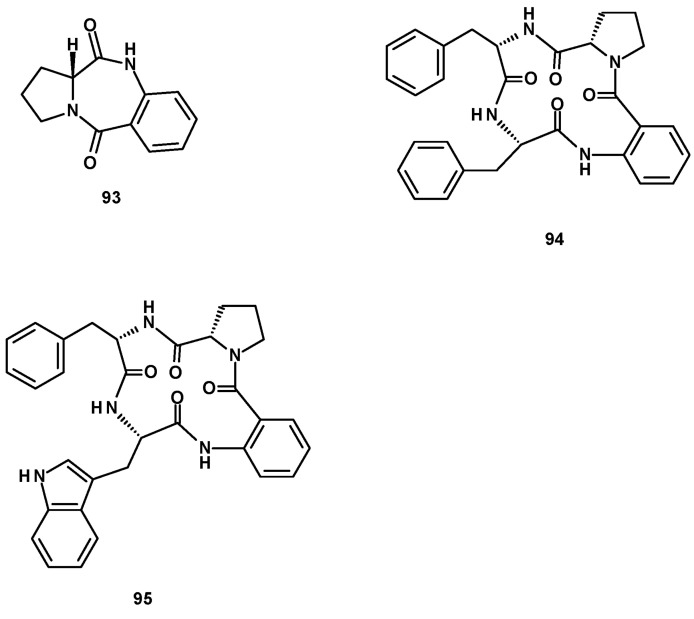
Structures of peptides **93**–**95**.

The previously reported dipeptide, (11a*R*)-2,3-dihydro-1*H*-pyrrolo [2,4-c][1,4]benzodiazepine-5,11 (10*H*,11a*H*)-dione (**93**), and two undescribed cyclic tetrapeptides, sartoryglabramides A (**94**) and B (**95**) (Figure 11) were isolated from a culture extract of the marine sponge-associated *N. glabra* KUFA 0702 [23]. The difference between **94** and **95** is that the Phe residue that linked with the anthranilic acid moiety in the former was replaced by Trp in the latter. The structures of both **94** and **95** were elucidated by extensive analysis of HRMS and 1D and 2D NMR data. The stereostructure of **94** was established by X-ray analysis using CuKα radiation, whereas the absolute configurations of the amino acid residues in **95** were determined by chiral HPLC analysis of its acidic hydrolysate, using appropriate D- and L-amino acid standards.

### 2.4. Terpenoids

**Figure 12:**
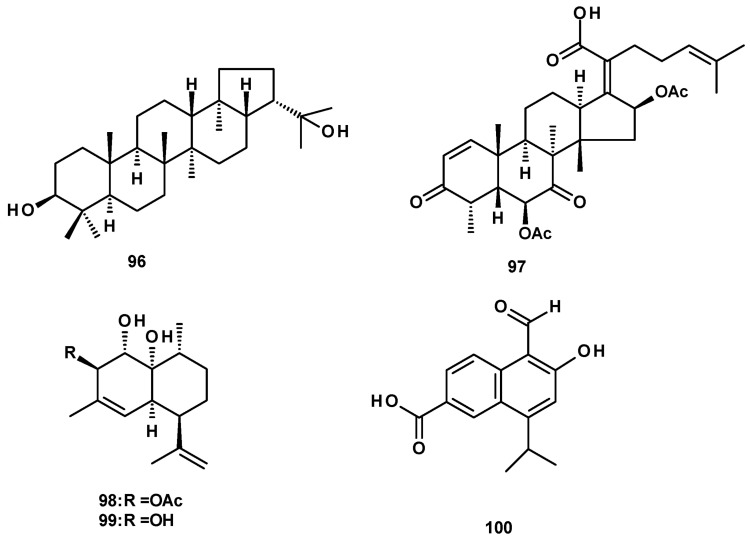
Structures of terpenoids **96**–**100**.

Terpenoids were not commonly found in *Neosartorya* species. The previously reported triterpene hopan-3β, 22-diol (**96**) (Figure 12), was isolated from a culture extract of the marine sponge-associated fungus *N. tsunodae* KUFC 9213 [30], whereas the nortriterpene, helvolic acid (**97**) (Figure 12), was very common and was reported from culture extracts of the soil-derived *N. fischeri* KUFC 6344 [25], as well as from the marine sponge-associated *N. tsunodae* KUFC 9213 and *N. fenelliae* KUFC 0811 [30].

The cadinene sesquiterpene (**98**) (Figure 12) was isolated from culture extracts of the soil-derived *N. pseudofischeri* KUFC 6422 [35] and *N. pseudofischeri* [41]. Compound **98** was previously obtained by the selective degradation of a natural product, CJ-12662 [52]. Compound **98** and its deacetyl derivative (**99**) were isolated, together with an aromatized cadinene, 5-formyl-6-hydroxy-8-isopropyl-2-naphthoic acid (**100**) (Figure 12), from a culture extract of the starfish-associated *N. pseudofischeri* [21].

### 2.5. Meroterpenoids

Meroterpenoids constitute a large group of specialized metabolites from *Neosartorya* species. They are structurally diverse and can be grouped according to the type of terpenoids, such as sesquiterpenes and diterpenes. Within the terpenoid class, they can be grouped according to a non-terpenoid moiety.

#### 2.5.1. Merosesquiterpenes

The first group of merosesquiterpenes is of the pyripyropenes and phenylpyripyropenes. In this group, the non-terpenoid moiety is derived from polyketides. The difference between these two groups is the presence of a pyridine ring in the former and a phenyl group in the latter. Several pyripyropenes with varying substituents have been reported from *N. fischeri* and *N. pseudofischeri*.

**Figure 13:**
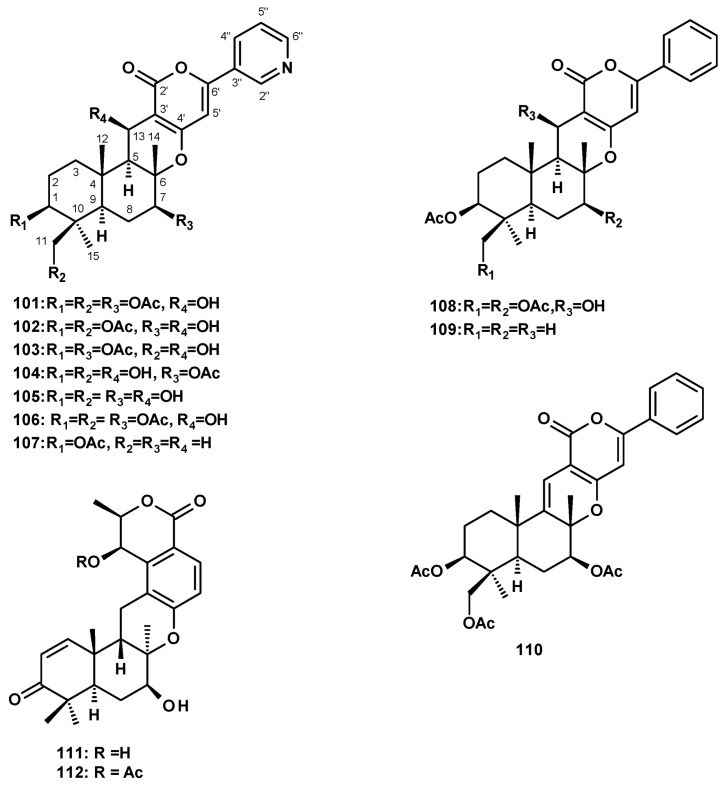
Structures of merosesquiterpenes **101**–**112**.

Pyripyropene A (**101**) (Figure 13) was reported from culture extracts of *N. fischeri* J80553 [27], *N. fischeri* NRRL 181 [33,49], the soil-derived *N. pseudofischeri* KUFC 6422 [35], the marine-derived *N. fischeri* [21], the sea-star-derived *N. pseudofischeri* [22], the soil-derived *N. pseudofischeri* [38] and *N. pseudofischeri* [41]. Several derivatives of pyripyropene A were also isolated from *N. fischeri* and *N. pseudofischeri*. In addition, 7-deacetylpyripyropene A (**102**) (Figure 13) was reported from culture extracts of the insect-derived *N. fischeri* [20], *N. fischeri* NRRL 181 [33], and the starfish-derived *N. pseudofischeri* [21], along with 11-deacetylpyripyropene A **(103**) (Figure 13) from the insect-derived *N. fischeri* [20], 1,11-dideacetylpyripyropene A (**104**) and 1,7,11-trideacetylpyripyropene A (**105**) (Figure 13) from *N. fischeri* NRRL 181 [33], and 13-dehydroxypyripyropene A (**106**) (Figure 13) from a starfish-derived *N. pseudofischeri* [21], while the unreported pyripyropene E (**107**) (Figure 13) was isolated from *N. fischeri* [41].

Two previously reported phenylpyripyropenes A (**108**) and B (**109**), and the unreported 5-olefin phenylpyripyropene A (**110**) (Figure 13) were also isolated from the starfish-derived *N. pseudofischeri* [21]. Finally, chrodrimanins A **(111**) and B (**112**) (Figure 13) were isolated from a culture extract of *N. glabra* CGMCC32286 [53].

**Figure 14:**
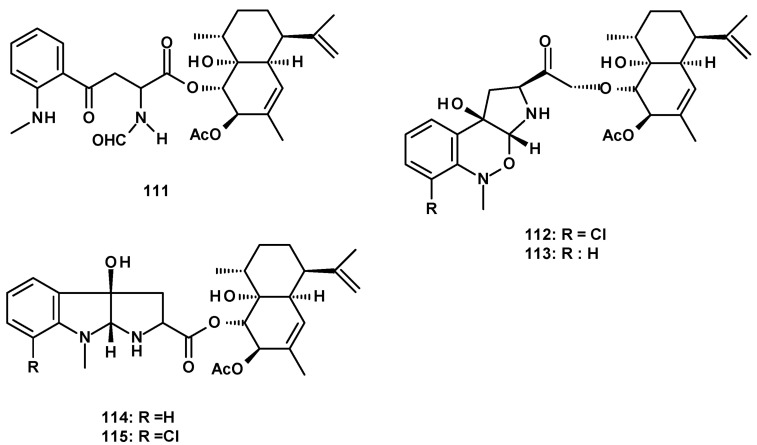
Structures of merosesquiterpenes **113**–**117**.

The second group of merosesquiterpenes consists of a cadinene sesquiterpene linked to aniline derivatives by an ester linkage. The previously described eurochevalierine (**113**) (Figure 14) was isolated from culture extracts of the soil-derived *N. pseudofischeri* KUFC6422 [35], the soil-derived *N. pseudofischeri* [38], *N. pseudofischeri* [41], and the soil-derived *N. hiratsukae* [39]. The previously reported merosesquiterpenes containing a pyrrolobenzoxazine moiety linked to a cadinene sesquiterpene, CJ-12662 (**114**), and CJ-12663 (**115**) (Figure 14) were isolated from the soil-derived *N. pseudofischeri* [38], *N. pseudofischeri* [41], and the soil-derived *N. hiratsukae* [39].

A cadinene ester of a pyrroloindole, *fischeri*ndoline (**116**) (Figure 14) was first reported from *N. fischeri* [41] and later from the soil-derived *N. pseudofischeri* [38], while its unreported 7-chloro derivative, 7-chloro*fischeri*ndoline (**117**) (Figure 14) was isolated from the soil-derived *N. hiratsukae* [39].

**Figure 15:**
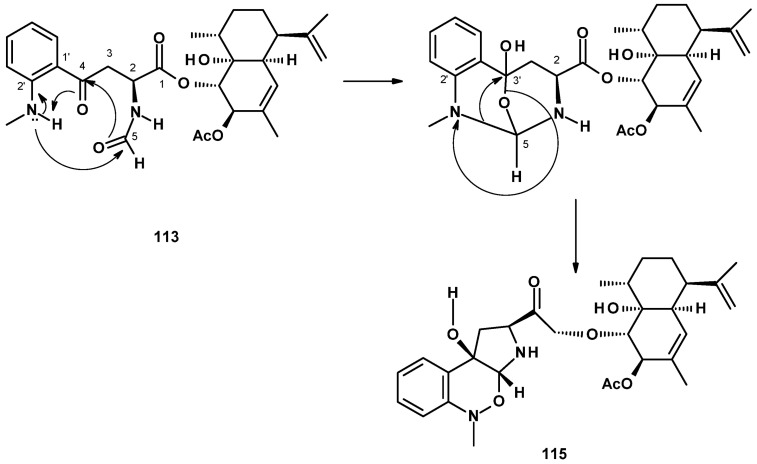
Formation of merosesquiterpenes **115** from **113**.

Compound **113** can be hypothesized as a biosynthetic precursor of **115,** as shown in Figure 15. The nucleophilic addition of the methylamino group on C-2′ to the aldehyde carbonyl (C-5), with a concomitant addition of the aldehyde oxygen to the carbonyl carbon attached to the benzene ring (C-4) in **113,** leads to the formation of an intermediate containing a bicyclic structure, linked by an ether bridge. Cleavage of the C-5-N and C-3-O bonds with the formation of C-3-N and N-O bonds leads to a pyrrolobenzoxazine moiety in **115**.

#### 2.5.2. Meroditerpenes

All the meroditerpenes isolated from members of the genus *Neosartorya* have polyketides with a variable number of acetate units in a non-terpenoid moiety. The most common diterpenoid moiety is tricyclic, but bicyclic, monocyclic, or even linear diterpenes have also been reported. They can be divided into three subgroups, according to the structure of the polyketide moiety.

##### Meroditerpenes Containing 2-Pyrone

**Figure 16:**
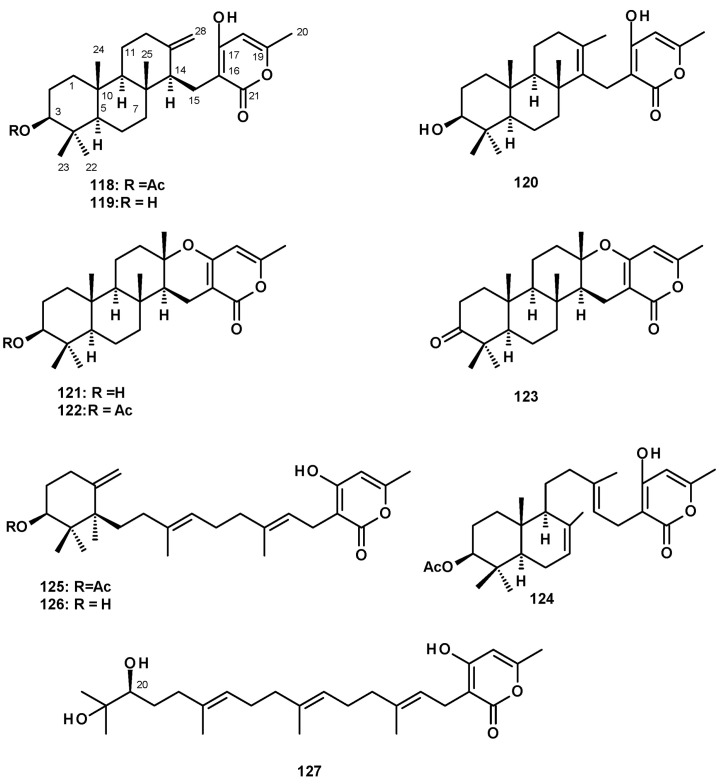
Structures of meroditerpenes **118**–**127**.

The most frequently isolated 2-pyrone-containing meroditerpene is aszonapyrone A (**118**) (Figure 16). Aszonapyrone A (**118**) consists of a tricyclic diterpene of a perhydrophenanthrene skeleton, linked to a 4-hydroxy-6-methyl-2*H*-pyran-2-one ring by a methylene bridge. Compound **118** was reported from a culture extract of the soil-derived *N. fischeri* KUFC 6433 and the marine-derived *N. laciniosa* KUFC 7896 [25], *N. fischeri* FO-5897 (cultured in sodden rice) collected from a soil sample from the city of Funabashi, Chiba, Japan [54], *N. fischeri* CGMCC3.5378 [26], the soil-derived *N. tatenoi* KKU-2NK23 [28], and the algicolous *N. takakii* KUFC 7898 [29]. Aszonapyrone B (**119**) (Figure 16), the deacetyl analog of **118**, was reported from *N. fischeri* CGMCC3.5378 [26], *N. fischeri* FO-5897 [54], the marine-derived *N. laciniosa* KUFC 7896 [25], and the soil-derived *N. tatenoi* KKU-2NK23 [28]. Sartorypyrone C (**120**) (Figure 16), an isomer of **119** with an endocyclic double bond instead of an exocyclic double bond, was reported from a culture extract of the marine sponge-associated *N. paulistensis* KUFC 7897 [46].

Another subgroup of 2-pyrone-containing meroditerpenes are the pentacyclic compounds, which have the diterpenoid moiety linked to a 2-pyrone ring through a dihydropyran ring. Chevalone A (**121**) (Figure 16) was isolated from the soil-derived *N. pseudofischeri* [38], while its acetate derivative, chevalone B (**122**) (Figure 16), was isolated from the soil-derived *N. siamensis* KUFC 6349 [42], the soil-derived *N. pseudofischeri* [38], the marine-derived *N. fenelliae* KUFA 0811 [30], the algicolous *N. takakii* KUFC 7898 [29], and the soil-derived *N. spinosa* KKU-1NK1 [44]. The unreported chevalone G (**123**), with a ketone group on C-3 of a diterpene moiety, and the unreported aszonapyrone G (**124**) (Figure 16), which contains a bicyclic diterpene moiety, were reported from the soil-derived *N. hiratsukae* [39], while a 2-pyrone-containing monocyclic meroditerpene, sartorypyrone A (**125**) (Figure 16) was first isolated from the soil-derived *N. fischeri* KUFC 6344 [25] and, later, from *N. fischeri* FO-5897 [54] and the plant endophytic *N. fischeri* JS0553 [27]. Sartorypyrone D (**126**) (Figure 16), a deacetylated derivative of **125**, was also isolated from *N. fischeri* JS0553 [27] and also from the soil-derived *N. hiratsukae* [39]. A 2-pyrone-containing meroditerpene with a linear diterpenoid bearing a vicinal diol function, sartorypyrone E (**127**) (Figure 16) was also isolated from *N. fischeri* JS0553. The absolute configuration of C-20 in **127** was established as 20*S* by ^1^H NMR analysis of its *S*- and *R*-MTPA esters [27].

##### Meroditerpenes Containing 4-Pyrone

**Figure 17:**
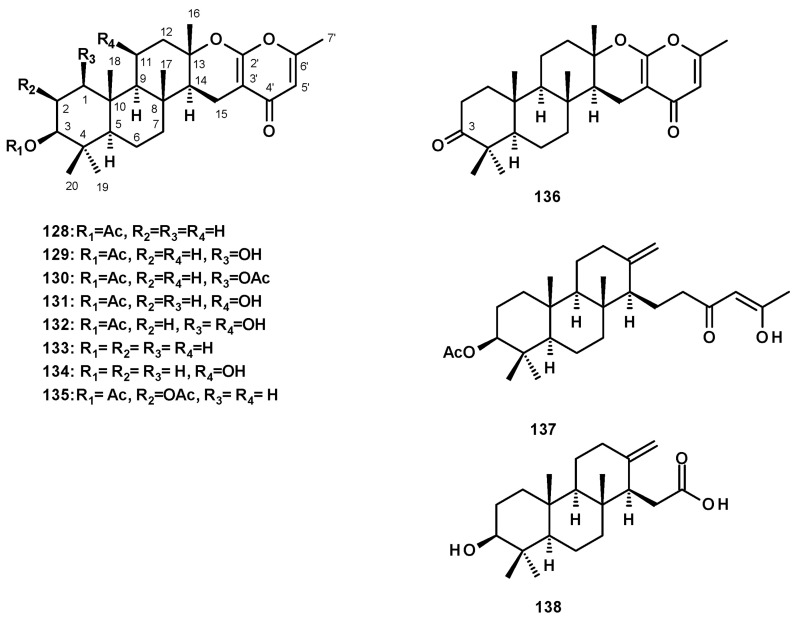
Structures of meroditerpenes **128**–**138**.

Meroditerpenes containing 4-pyrone, isolated from *Neosartorya* species, consist of a tricyclic diterpene skeleton, linked to a 4-pyrone ring through a dihydropyran moiety. Chevalone C (**128**) (Figure 17) was reported from a culture extract of *N. siamensis* KUFC 6349 [42], the marine-derived *N. siamensis* KUFA 0017 [43], the soil-derived *N. spinosa* KKU-1NK1 [44], the marine-derived *N. tsunodae* KUFC 9213 [30], the soil-derived *N. pseudofischeri* [38], and the soil-derived *N. hiratsukae* [39].

Several derivatives of chevalone C (**128**) have been reported from *Neosartorya* species. The unreported 1-hydroxychevalone C (**129**), 1-acetoxychevalone C **(130**), 11-hydroxychevalone C (**131**), 1,11-dihydroxychevalone C (**132**), and the previously reported chevalone E **(133**) (Figure 17) were isolated from a culture extract of the soil-derived *N. spinosa* KKU-1NK1 [44]. Compounds **131** and **133** were also isolated from *N. pseudofischeri* [38] and the soil-derived *N. hiratsukae* [39], while the unreported 11-hydroxychevalone E (**134**) (Figure 17) was also isolated from the soil-derived *N. pseudofischeri* [38]. The previously described sartorypyrone B (**135**) (Figure 17) was also isolated from the marine-derived *N. tsunodae* KUFC 9213 [25] and the marine-derived *N. fenelliae* KUFA 0811 [30]. The undescribed chevalone F (**136**), with a ketone group on C-3, was isolated from *N. pseudofischeri* [38] (Figure 17).

##### Meroditerpenes Containing a Linear Polyketide Moiety

The undescribed sartorenol (**137**) (Figure 17), a meroditerpene consisting of a tricyclic diterpene with an unusual (4*Z*)-5-hydroxy-3-oxohex-4-en-1-yl substituent, was isolated from a culture extract of the marine-derived *N. takakii* KUFC 7898 [29], while the unreported tatenoic acid (**138**) (Figure 17), the substituent of which is a carboxymethyl group, was isolated from the soil-derived *N. tatenoi* KKU-2NK23 [28].

### 2.6. Sterols and Sterones

**Figure 18:**
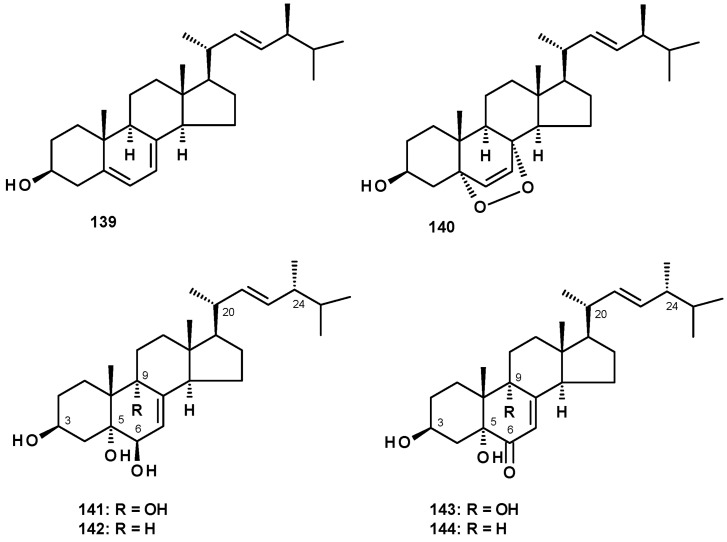
Structures of sterols **139**–**144**.

Ergosterol (**139**) and its 5,8-endoperoxide (**140**) (Figure 18) were isolated from *N. pseudofischeri* CGMCC 3.5378 [31]. Compound **139** was also reported from *N. fischeri* NRRL 181 [33] and *N. tatenoi* KKU-2NK23 [28].

Ergosterol analogs, viz. (22*E*, 24*R*)-ergosta-7, 22-dien-3β, 5α, 6β, 9α-tetraol **(141**), (22*E*, 24*R*)-ergosta-7,22-dien-3β, 5α, 6β-triol (**142**), 3β, 5α,9α-trihydroxy (22*E*, 24*R*)-ergosta-7,22-dien-6-one (**143**) and 3β, 5α-dihydroxy (22*E*, 24*R*)-ergosta-7,22-dien-6-one (**144**) (Figure 18) were isolated from *N. fischeri* NRRL 181 [33]. Compound **144** was also isolated from the marine-derived *N. tsunodae* KUFA 0811 [30].

**Figure 19:**
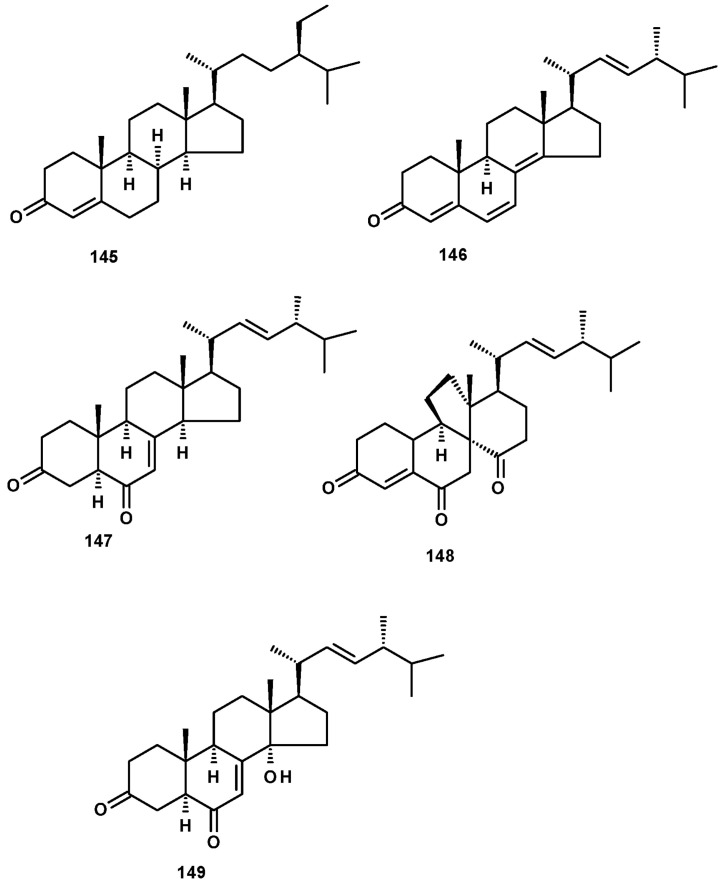
Structures of sterones **145**–**149**.

Sterones have been also reported from members of the genus *Neosartorya*. Sitostenone (**145**), ergosta-4,6,8(14),22-tetraen-3-one (**146**), cyathisterone (**147**) and dankasterone A (**148**) (Figure 19) were reported from the marine-derived *N. fenelliae* KUFA 0811 [30], while (14α,22*E*)-14-hydroxy-ergosta-7,12-dien-3,6-dione (**149**) (Figure 19) was reported from *N. fischeri* NRRL 181 [33].

### 2.7. Polyketides

**Figure 20:**
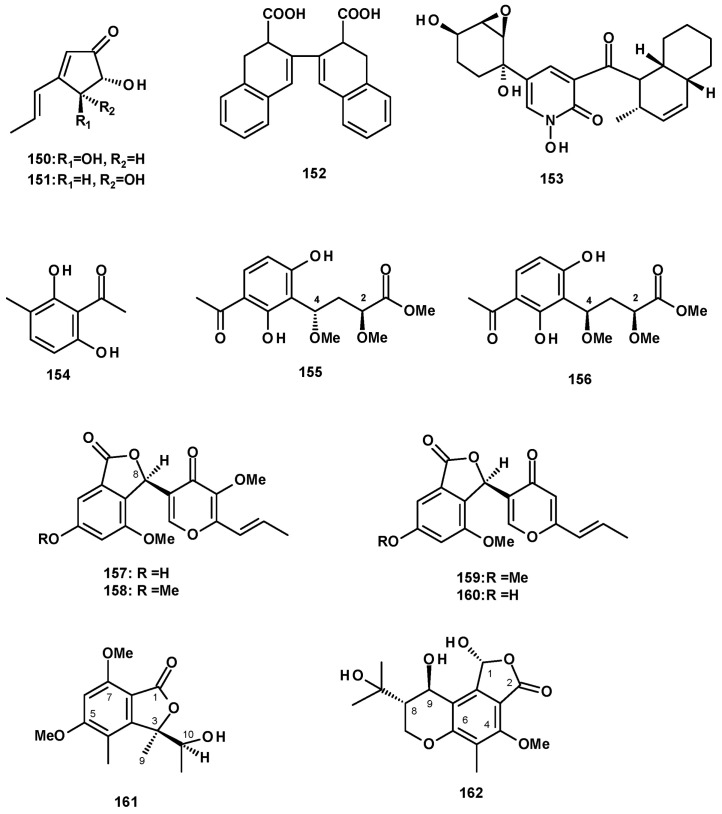
Structures of polyketides **150**–**162**.

Secondary metabolites derived from polyketides, which have diverse structural features, are the most abundant group produced by the *Neosartorya* species. Two previously reported cyclopentenone derivatives, terrein **(150**) and isoterrein (**151**) (Figure 20), were isolated from a culture extract of *N. fischeri* IFM 52672 cultured in moist rice [24]. Fischeacid (**152**) (Figure 20), a bis-decalin polyketide, was isolated from a culture extract of the marine-derived *N. fischeri* 1008F1 [55]. *Fischeri*n (**153**) (Figure 20), possessing a decalin scaffold linked to a hydroxypyridone moiety by a carbonyl group, was first reported from *N. fischeri* var. *fischeri* CBM-FA-0156 [34] and, later, from a culture extract of *N. fischeri* JS0553 [27]. Fujimoto et al. proposed its biogenesis as being derived from Phe and a heptaketide [34]. A great number of microbial secondary metabolites containing decalin motif, with structural diversity and relevant biological activity, has been reported. Li et al. have presented an excellent review on natural products containing the decalin motif in the form of microorganisms [56].

Acetophenones were also reported from some species of *Neosarirya*. First, 2,6-Dihydroxy-3-methylacetophenone (**154**) (Figure 20) was isolated from a culture extract of the soil-derived *N. siamensis* KUFC 6349 [42], as well as from the marine-derived *N. siamensis* KUFA0017 [43]. The undescribed 2*S*, 4*S*-spinosate (**155**) and 2*S*, 4*R*-spinosate (**156**) (Figure 20) were isolated from a culture extract of *N. spinosa* KKU-1NK1. The absolute configurations at C-2 and C-4 in both compounds were established by the comparison of calculated and experimental ECD spectra [44].

Another group of polyketides comprises the benzofuranone derivatives. The unreported neosarphenol A (**157**), and the previously reported methoxyvermistatin (**158**), vermistatin (**159**), and 6-demethylvermistatin (**160**) (Figure 20) were isolated from a culture extract of *N. glabra* CGMCC32286. The absolute configuration at C-8 in **157** was determined via a comparison of the sign of its optical rotation with that of the known **158** [53]. The undescribed quadricinctone A (**161**) (Figure 20) was isolated from a solid rice culture extract of the marine sponge-associated fungus *N. quadricincta* KUFA0081. The absolute configurations at C-3 and C-10 were established as 3*R*, 10*S* by X-ray analysis using CuKα radiation [57]. A chromanol derivative (**162**) (Figure 20) was isolated from a culture extract of the marine sponge-associated fungus *N. tsunodae* KUFC 9213. The structure of the compound was elucidated via the analysis of HRMS and 1D and 2D NMR spectral data. The absolute configurations at C-1, C-8 and C-9 were determined as 1*R*, 8*S*, and 9*R* by X-ray analysis using CuKα radiation [30].

**Figure 21:**
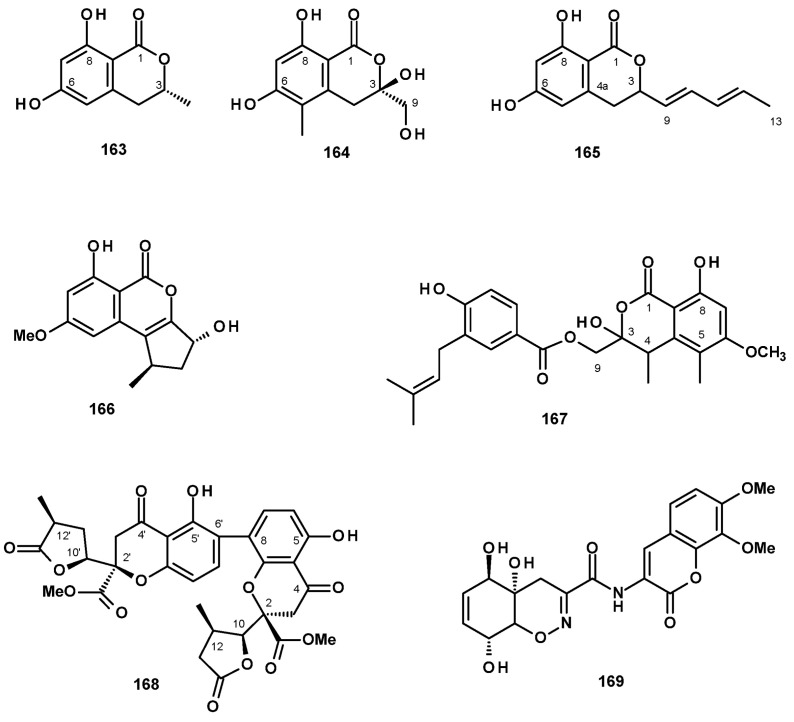
Structures of **163**–**169**.

Isochromanones have been reported from both terrestrial and marine-derived *Neosartorya* species. (*R*)-6-Hydroxymellein (**163**) (Figure 21) was reported from a culture extract of the algicolous fungus *N. takakii* KUFC 7898 [29], as well as from a solid rice culture extract of the marine sponge-associated *N. spinosa* KUFA 1047 [58]. The undescribed quadricinctone C (**164**) (Figure 21) was isolated from a culture extract of the marine sponge-associated fungus *N. quadricincta* KUFA0081. The absolute configurations at C-3 and C-4 were established as 3*S*, 4*R* by X-ray analysis using CuKα radiation [57]. The unreported 6,8-dihydroxy-3-(1*E*,3*E*)-penta-1,3-dien-1-yl) isochroman-1-one (**165**) (Figure 21) was isolated from a culture extract of the starfish-derived *N. pseudofischeri*. Its structure was established by the interpretation of HRMS and 1D and 2D NMR data; however, their absolute configuration at C-3 was not determined [21]. The previously reported phialophoriol (**166**) (Figure 21) was isolated from a culture extract of *N. glabra* CGMCC32286 [53]. The unreported prenyl 4-hydroxybenzoic acid ester of a dihydrochromone, PF1223 (**167**) (Figure 21), was isolated from a culture extract of *N. quadricincta* strain PF1223, which was obtained from the Meiji Seika Kaisha collection and cultured in a solid medium containing raw rice and soybean meal. The structure of **167** was established by 1D and 2D NMR spectral analysis and HRMS data; however, the absolute configurations of the stereogenic carbons C-3 and C-4 were not determined [59].

The undescribed dihydrochromone dimer, paecilin E (**168**) (Figure 21), was isolated from the marine sponge-associated *N. fenelliae* KUFA 0811. The structure of **168** was established based on an extensive analysis of 1D and 2D NMR spectra and HRMS data. The absolute configurations of the stereogenic carbons, C-2, C-2′, C-10, C-10′, C-11, and C-11′ were determined as 2*R*, 2′*R*, 10*S*,10′*S*, 11*R*, 11′*R* by X-ray analysis using CuKα radiation [30].

The previously reported trichodermamide A (**169**) (Figure 21), whose structure consists of a coumarin nucleus linked to a tetrahydro 1,2-benzoxazine moiety through an amide linkage, was isolated from a culture extract of the starfish-derived *N. pseudofischeri* [21].

**Figure 22:**
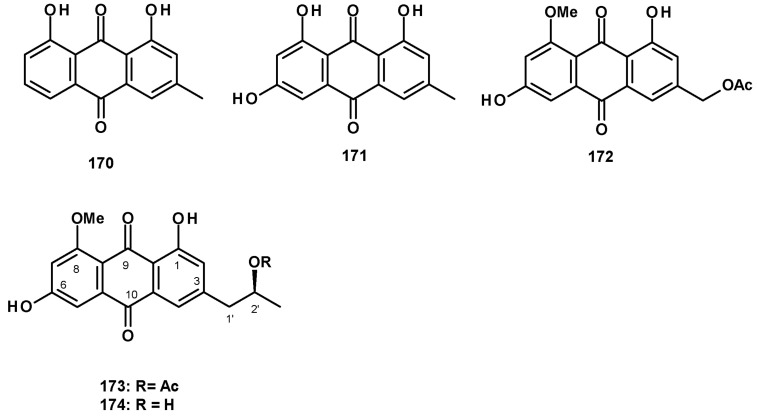
Structures of **170**–**174**.

The previously reported anthraquinones, chrysophanol (**170**) and emodin (**171**) (Figure 22), were isolated from a culture extract of the marine-derived *N. fischeri* 1008F1 [55]. The previously reported acetylquestinol (**172**) was isolated as a 1:3 mixture with the undescribed acetylpenipurdin A **(173**), together with the previously reported penipurdin A **(174**) (Figure 22), from a culture extract of the marine sponge-associated *N. spinosa* KUFA1047 [58].

**Figure 23:**
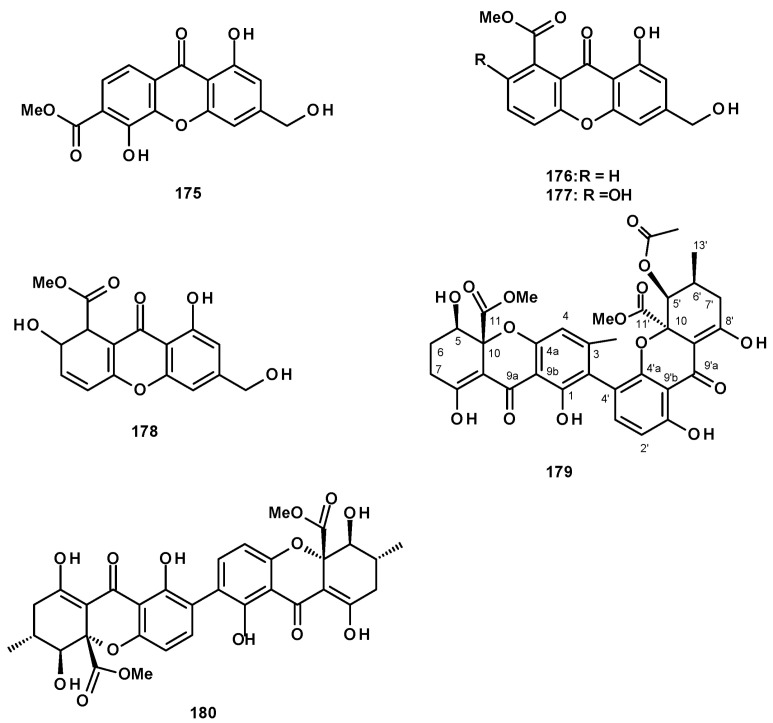
Structures of **175**–**180**.

Polyhydroxylated xanthones and bis-xanthone derivatives were also reported from *Neosartorya* species, especially *N. fischeri*. The unreported fischexanthone (**175**) was isolated, together with the previously reported sydowinins A (**176**) and B (**177**), and AGI-B4 (**178**) (Figure 23) from a culture extract of *N. fischeri* 1008 F1 [55]. The undescribed bis-xanthone derivative, neosartorin (**179**) (Figure 23), was isolated from a liquid culture extract of *N. fischeri*, isolated from sediment from the River Vah in Slovakia. The structure of the compound was elucidated by extensive analysis of HRMS and 1D and 2D NMR data. The relative stereochemistry of **179** was determined on the basis of ^1^H-^1^H coupling constants of *J*_H-5/H-6ax_ (2.0 Hz) and *J*_H-5/H-6eq_ (4.0 Hz), *J*_H-6′/H-7′ax_ (10 Hz), as well as by observation of the nuclear Overhauser effects (NOEs) between H-2′ of the carboxymethyl group and OH-1 and OH-8, as well as between the methyl protons of COOMe on C-5′ and H-3 [60]. The previously reported secalonic acid A (**180**) (Figure 23) was isolated from a culture extract of the marine sponge-associated *N. fenelliae* KUFA 0811 [30].

**Figure 24:**
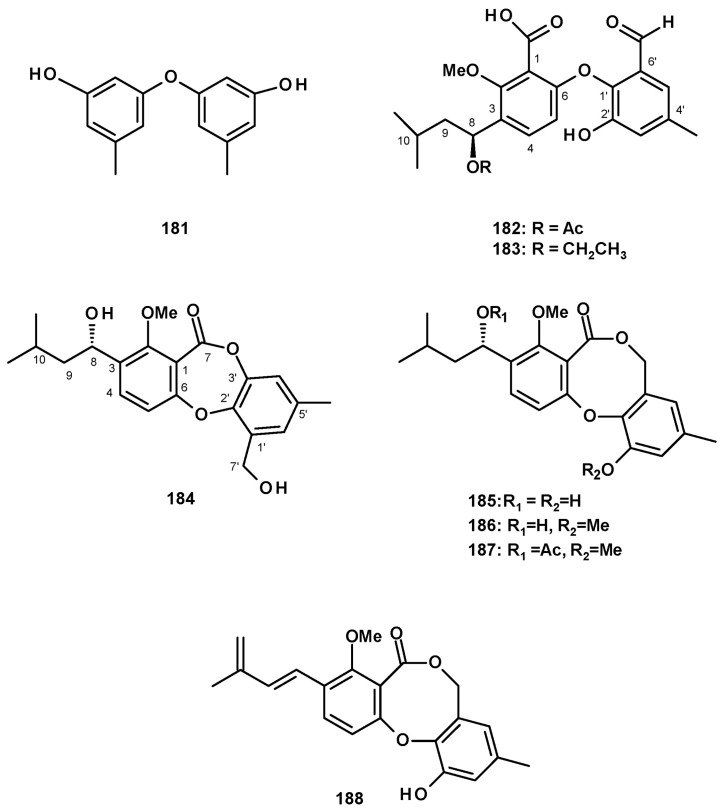
Structures of **181**–**188**.

Another group of polyketides is the biphenyl ethers and their derivatives. The previously described diorcinol (**181**) (Figure 24) was isolated from a culture extract of the soil-derived *N. hiratsukae* [39]. The previously reported tenellic acid (**182**), the undescribed neospinosic acid (**183**) and spinolactone (**184**), and the previously reported vermixocin A (**185**) (Figure 24) were isolated from a culture extract of the marine sponge-associated *N. spinosa* KUFA 1047 [58]. Since the absolute configuration at C-8 in **182** had not been established, de Sá et al. [58] determined the absolute configuration of C-8 in **182** as 8*S* by the comparison of its calculated and experimental ECD spectra. The structures of the unreported **183** and **184** were established by extensive analysis of their HRMS and 1D and 2D NMR data. The absolute configuration at C-8 in both compounds was determined as 8*S* by comparison of their calculated and experimental ECD spectra.

Two previously reported penicillide (**186**) and purpactin A (**187**) were isolated, together with the unreported neosarphenol B (**188**) (Figure 24), from a culture extract of *N. glabra* CGMCC32286 [53].

**Figure 25:**
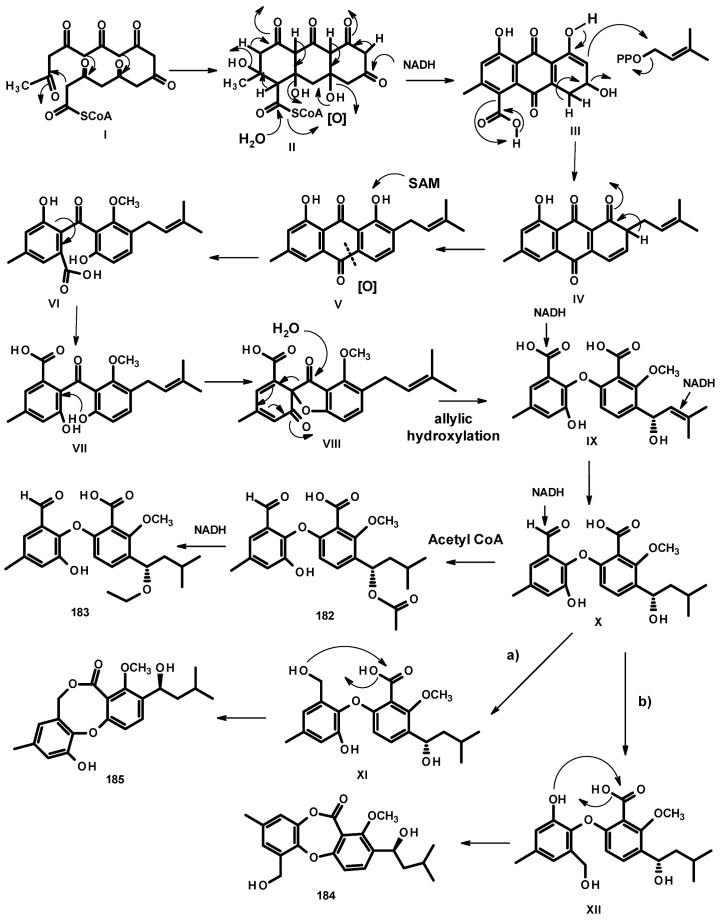
Proposed biosynthetic pathways to **182**–**185**.

In their study, de Sá et al. [58] proposed the biosynthetic relationship of **182**–**185**, as depicted in Figure 25. The biosynthesis of **182**–**185** starts with a cyclization of the octaketide (**I**) to form the intermediate **II**. Enolization, the reduction of a carbonyl group, and hydrolysis of acetyl CoA in **II** lead to the formation of the intermediate **III**. Decarboxylation, enolization, and prenylation by dimethylallyl pyrophosphate (DMAPP) give the intermediate **IV**, which, after enolization, gives rise to the prenylated anthraquinone **V**. Methylation of the phenolic hydroxyl group and oxidative cleavage of the ring of the anthraquinone intermediate **V** leads to the formation of **VI**. The nucleophillic addition of **VI** (=**VII**) by a hydroxyl group leads to **VIII**, which, after the addition of H_2_O to the carbonyl group with cleavage of the bond between the benzene ring and the carbonyl group, followed by the enzymatic allylic oxidation of the prenyl group, leads to the formation of the biphenyl ether **IX**. Reduction of the double bond of the prenyl group in **IX** gives **X**. Acetylation of the hydroxyl group of the side chain (C-8) gives rise to **182,** which, after oxidation of the carbonyl of the acetyl group, leads to the formation of **183**.

The formyl group in **X** can be reduced to a primary alcohol in **XI** (=**XII**). Esterification of the carboxyl group by a primary alcohol in **XI** leads to the formation of **185**, while esterification by a phenolic hydroxyl group in **XII** leads to the formation of **184**.

**Figure 26:**
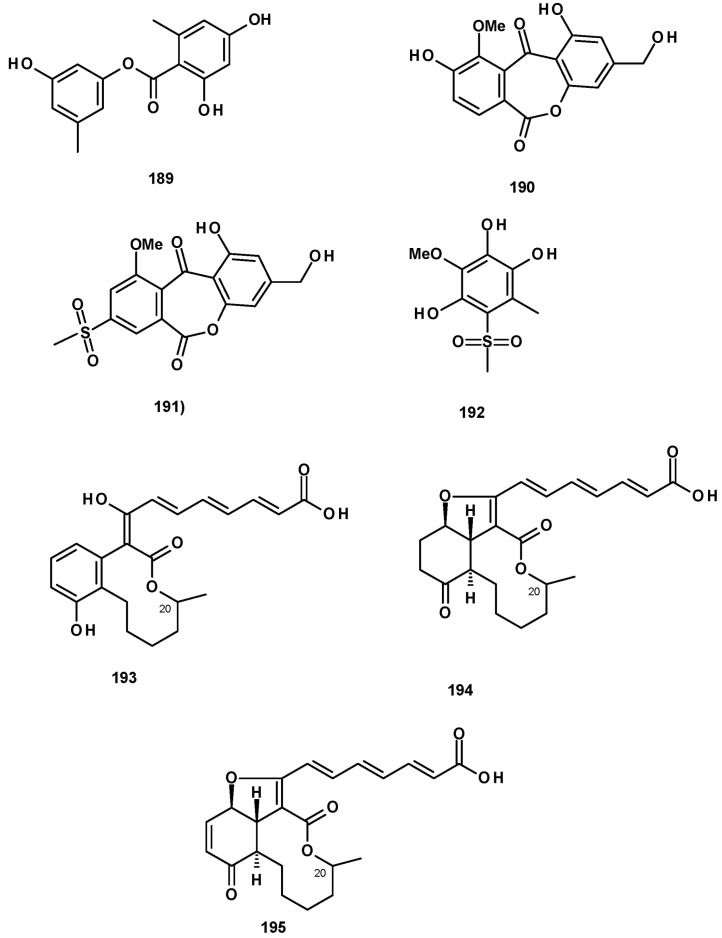
Structures of **189**–**195**.

Polyketides also originate hydroxybenzoic acid esters and lactones. 2,4-Dihydroxy-6-methylbenzoic acid ester (**189**) (Figure 26) was isolated from culture extracts of the soil-derived *N. pseudofischer*i KUFC 6422 [35], *N. pseudofischeri* [38], and *N. hiratsukae* [39]. A previously reported biphenyl lactone (**190**) and its unreported methylsulfonyl analog, neosartoryone A (**191**), and 3-methoxy-6-methyl-5-(methylsulfonyl)benzene-1,2,4-triol (**192**) (Figure 26) were isolated from a liquid culture extract of *N. udagawae* HDN13-313 with the addition of 5-azacytidine in the culture medium. It was proved that the methylsulfonyl substituent in **191** and **192** originated from dimethyl sulphoxide (DMSO), which was used as a solvent to dissolve 5-azacytidine [61].

Glabramycins A (**193**), B (**194**), and C (**195**) (Figure 26) are macrocyclic lactones, isolated from a solid culture extract of *N. glabra* (strain MF7030, F-155,700) obtained from a hot-water-pasteurized soil that was collected in Valdefresno Province in Spain. The structures of the compounds were elucidated by 1D and 2D NMR and HRMS data. However, the absolute configuration at C-20 was not determined [62].

### 2.8. Benzoic Acid Derivatives

**Figure 27:**
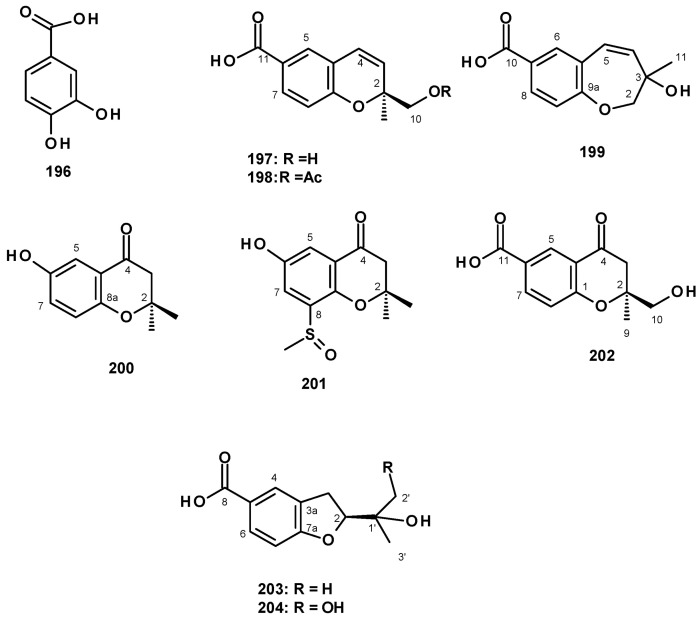
Structures of **196**–**204**.

Although secondary metabolites originating from benzoic acid are not ubiquitous in fungi such as indole alkaloids, meroterpenoids, and polyketides, some of them have been reported sporadically. The previously reported 3,4-dihydroxybenzoic acid (**196**) (Figure 27) was isolated from a culture extract of the marine-derived *N. fischeri* 1008F1 [55].

The unreported benzoic acid derivatives, quadricinctapyan A (**197**), quadricinctapyan B **(198**) and quadricinctoxepine (**199)**, the previously reported 2,3-dihydro-6-hydroxy-2,2-dimethyl-4*H*-1-benzopyran-4-one (**200**), and the undescribed quadricinctone B (**201**), quadricinctone D **(202**), quadricinctafuran A **(203**), and quadricintafuran B (**204**) (Figure 27) were isolated from a solid rice culture extract of the marine sponge-associated *N. quadricincta* KUFA 0081 [57]. The structures of the compounds were established by extensive analysis of 1D and 2D NMR spectra and HRMS data. The absolute configurations of the stereogenic carbons, i.e., C-2 in **197**, **202,** and **203** were established as 2*S*, 2*S,* and 2*R*, respectively, by X-ray analysis using CuKα radiation. Moreover, the Ortep view also revealed the configuration of the sulfoxide group in **201** as *R*. However, the configuration of C-3 in **199** and C-1′ in **204** were still undetermined. It is worth mentioning that marine natural products with methyl sulfoxide substituents, such as in **201,** are not very common.

**Figure 28:**
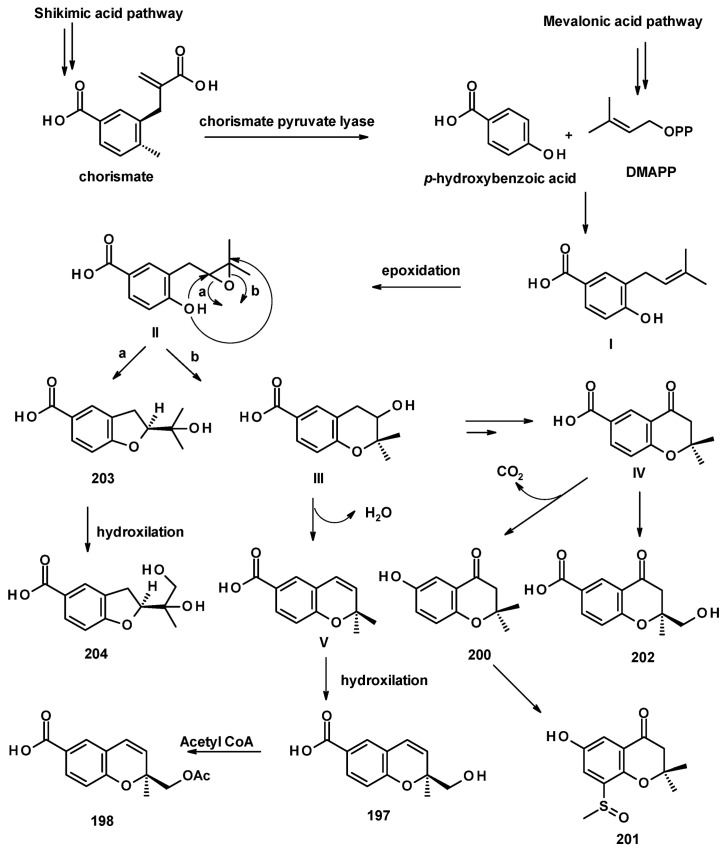
Proposed biosynthetic pathways for **197**, **198**, **200**–**204**.

The biosynthetic pathways for **197**–**204** were proposed to be of mixed origin, i.e., shikimic acid and mevalonic acid pathways, similar to that proposed for fomannoxin [63]. The biosynthetic pathways start with the formation of *p*-hydroxybenzoic acid by elimination of a pyruvate moiety from chorismate by chorismate pyruvate lyase. The prenylation of *p*-hydroxybenzoic acid by DMAPP leads to the formation of **I**, which, after epoxidation of the double bond of the prenyl group, forms **II**. Nucleophilic substitution of the epoxide by a phenolic hydroxyl group gives rise to **203** (route **a**) or **III** (route **b**). Hydroxylation of one of the methyl groups of the prenyl side chain in **203** leads to the formation of **204**. Another pathway is the dehydration of **III**, resulting in the formation of **V** which, upon hydroxylation of one of the methyl groups, leads to the formation of **197**. On the other hand, **III** can undergo dehydration, followed by regiospecific hydration and oxidation to give **IV**, which can be either hydroxylated at one of the methyl groups to give **202** or undergoes decarboxylation and aromatic hydroxylation to give **200**. The introduction of a methyl sulphonyl group to the benzene ring results in the formation of **201** (Figure 28) [57].

**Figure 29:**
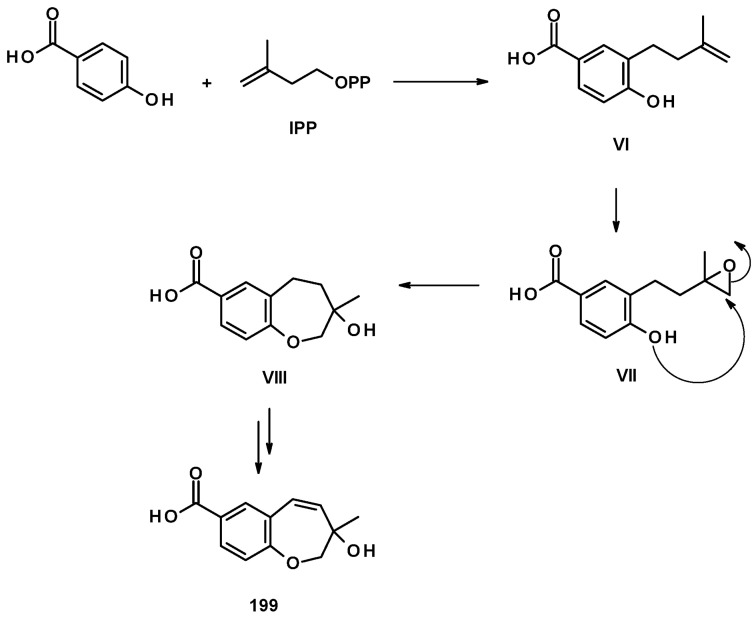
Proposed biosynthetic pathway for **199**.

Compound **199** is also derived from *p*-hydroxybenzoic acid but uses isopentenyl pyrophosphate (IPP) as a prenylating agent to form **VI**. The epoxidation of the terminal double bond of the isopentenyl group gives **VII,** which, upon the nucleophilic substitution of the epoxide by a phenolic hydroxyl group, leads to the formation of a hydroxyoxepine ring in **VIII**. Further desaturation of the hydroxyoxepine ring gives rise to **199** (Figure 29) [57].

### 2.9. Nucleosides

**Figure 30:**
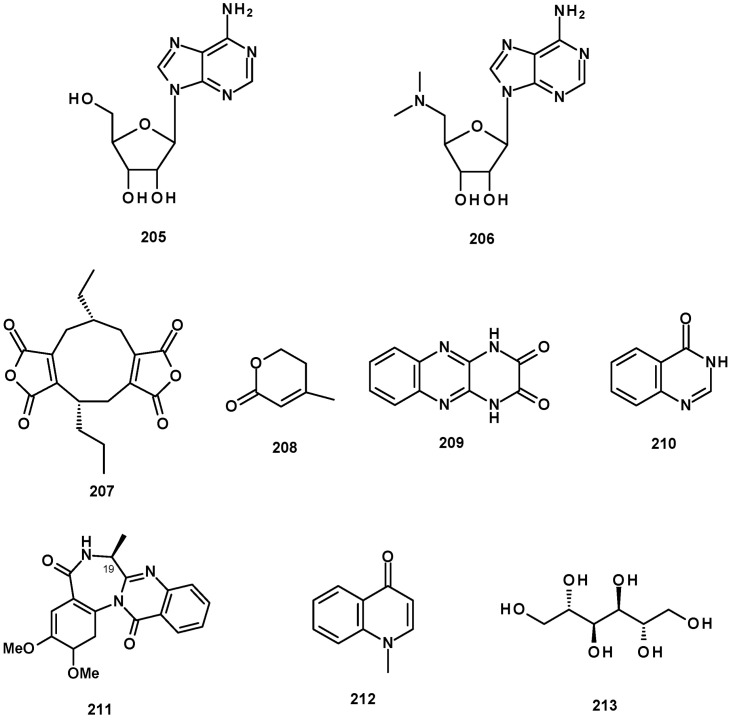
Structures of **205**–**213**.

Only two nucleosides, adenosine (**205**) and 5′-deoxy-5′-methylaminoadenosine (**206**) (Figure 30), were reported from the marine-derived *N. fischeri* 1008 F1 [55].

### 2.10. Miscellaneous

Nanodrides are fungal metabolites containing a nine-membered ring fused to one or two maleic anhydride moieties. Although several nanodrides have been reported from the cultures of many fungal species, only byssochlamic acid (**207**) (Figure 30) was isolated from cultures of the marine sponge-associated *N. fenelliae* KUFA 0811 and *N. tsunodae* KUFC 9213 [30].

Dehydromevalonic acid (**208**) and lumichrome (**209**) (Figure 30) were also isolated from the marine sponge-associated *N. tsunodae* KUFC 9213 [30]. Lumichrome is a derivative of the vitamin riboflavin and was found to activate the LasR quorum-sensing (QS) receptor. LasR normally recognizes the *N*-acyl homoserine lactone (AHL) signal. Amino acid substitutions in the LasR residues required for AHL binding altered the responses to both AHLs and lumichrome/riboflavin. Bacteria, plants, and algae commonly secrete riboflavin and/or lumichrome, raising the possibility that these compounds could serve as either QS signals or as interkingdom-signal mimics capable of manipulating QS in bacteria with a LasRlike receptor [64]. It is of note that, although lumichrome is commonly found in bacteria, plants, and algae, it is rarely reported from fungi.

In addition, 4(3*H*)-quinazolinone (**210**) (Figure 30) was isolated from the marine sponge-associated *N. paulistensis* KUFC 7897 [46]. It is interesting to note that although many quinazolinone-containing indole alkaloids have been isolated from many *Neosartorya* species, this is the first isolation of a simple 4(3*H*)-quinazolinone from the fungus of the genus *Neosartorya*.

A 4(3*H*)-quinazolinone-containing non-indole alkaloid, 5,6-dimethoxycircumdatin C (**211**) (Figure 30), was isolated from the insect-derived *N. fischeri* TJ403-CA8. The structure of the compound was established by the analysis of HRMS and 1D and 2D NMR data. The absolute configuration at C-19 was determined as 19*S* by X-ray analysis using CuKα radiation [20].

Finally, 1-Methyl-4-quinolone (**212**) (Figure 30) was isolated from a culture extract of *N. hiratsukae* [39], whereas mannitol (**213**) (Figure 30) was reported from *N. tatenoi* KKU-2NK23 [28].

## 3. Biological Activity of Secondary Metabolites Produced by Fungi of the Genus *Neosartorya*

Some compounds isolated from members of the genus *Neosartorya* were tested for several biological/pharmacological activities, mostly in vitro. Like all other natural products, a majority of the compounds isolated from *Neosartorya* species were tested for in vitro anticancer/cytotoxic and antimicrobial activities. For practical aspect, they can be divided as follows:

### 3.1. Anticancer Activity/Cytotoxicity

Eamvijarn et al. have evaluated aszonalenin (**8**), acetylaszonalenin (**9**), 1-formyl-5-hydroxyaszonalenin (**11**) (Figure 2), 13-oxofumitremorgin B (**25**) (Figure 3), aszonapyrone A (**118**) and sartorypyrone A (**125**) (Figure 16) isolated from the soil-derived *N. fischeri* KUFC 6344, aszonapyrone B (**119**) (Figure 16) isolated from the marine-derived *N. laciniosa* KUFC 7896, and sartorypyrone B (**135**) (Figure 17) isolated from the marine-derived *N. tsunodae* KUFC 9213, for their capacity to inhibit the in vitro growth of MCF-7 (breast adenocarcinoma), NCI-H460 (non-small cell lung cancer), and A375-C5 (melanoma) cell lines, by the protein binding dye sulforhodamine B (SRB) method. Compound **119** was the most active, exhibiting strong growth inhibitory activity against the three cell lines, with GI_50_ values of 13.6, 11.6, and 10.2 µM for MCF-7, NCI-H460, and A375-C5, respectively, while **118** was inactive at the highest concentration tested (150 µM). Compound **135** also exhibited strong growth inhibitory activity, although less actively than **118**, with GI_50_ values of 17.8, 20.5, and 25.0 µM for MCF-7, NCI-H460, and A375-C5, respectively. Interestingly, **125**, which possesses a monocyclic diterpene core, was more selective, exhibiting similar inhibitory activity to **135** against A375-C5 (GI_50_ = 1.5 µM), but less active against MCF-7 (GI_50_ = 46.3 µM) and NCI-H460 (GI_50_ = 37.3 µM) cell lines. On the other hand, all the three aszonalenin derivatives, **8**, **9,** and **11**, were found to be inactive against all the three cell lines at the highest concentration tested (150 µM), whereas **25** exhibited only weak inhibitory activity against all the three cell lines, with GI_50_ values of 115.0, 123.3, and 68.6 µM, for MCF-7, NCI-H460, and A375-C5, respectively [25].

A hydroxylated xanthone, AGI-B4 (**178**) (Figure 23), isolated from a culture extract of *N. fischeri* 1008 F1, exhibited inhibition of the proliferation of a human gastric cancer cell line SGC-7901, with an IC_50_ value of 0.29 mmol/L, and hepatic cancer cell line BEL-7404, with an IC_50_ value of 0.31 mmol/L, by the MTT (3-(4,5-dimethylthiazol-2-yl)-2,5-diphenyltetrazolium bromide) assay. The positive control, 5-fluorouracil, showed 85.6% and 83% cell proliferation inhibition against SGC-7901 and BEL 7404, respectively [55].

Fiscalin B (**3**) (Figure 1) and two anellated indoles **78** and **79**, isolated from a culture extract of the marine-derived *N. fischeri*, exhibited cytotoxicity by apoptosis against HL-60 (human leukemia) cells with IC_50_ values of 82.3, 90.0, and 8.88 µM, respectively [19].

Eamvijarn et al. evaluated the in vitro growth inhibitory activity of the cadinene sesquiterpene (**98**) (Figure 12) and eurochevalierine (**113)** (Figure 14), isolated from a culture extract of the soil-derived *N. pseudofischeri* against Hs683 (human glioblastoma), U373 (human glioblastoma), A549 (non-small cell lung cancer), MCF-7 (breast cancer), OE21 (esophageal cancer) and SKMEL28 (melanoma) cell lines. Compound **113** displayed in vitro anticancer activity in the range displayed by etoposide and carboplatin, whereas **98** exhibited less activity than **113** but was similar to that of carboplatin. Computer-assisted phase-contrast microscopy demonstrated that **113** displayed cytostatic and not cytotoxic effects in human U373 and A549 cells. Moreover, flow cytometry analysis confirmed the lack of cytotoxicity of **113,** since no pro-apoptotic effects were observed with **113** in U373 and A549 cells. Flow cytometry analysis also showed that **113** did not modify cell cycle kinetics, such as the distribution of cells into the G1, S, and G2 phases of the cell cycle of A549 and U373 cells [35].

Masi et al. evaluated the in vitro growth inhibitory effect of *fischeri*ndoline (**116**) (Figure 14), isolated from a culture extract of *N. pseudofischeri* strain CBS 404.67, in six human and one mouse cancer cell lines, viz. A549, Hs683, MCF-7, SKMEL28, U373, B16F10 (melanoma). However, **116** was found to exhibit a similar activity to that of **113** and pyripyropene E (**107**) (Figure 13). Curiously, **113**, **116**, and **107** displayed less potent activity than gliotoxin (**49**) (Figure 6) in the tested cell lines [41].

Liang et al. screened the cytotoxic effects of 1,2,3,4-tetrahydro-2-methyl-3-methylene-1,4-dioxopyrazino [1,2-*a*]indole (**46**), 1,2,3,4-tetrahydro-2-methyl-1,3,4-trioxopyrazino [1,2-*a*]indole (**47**), gliotoxin (**49**), acetylgliotoxin (**50**), bis (dethio)bis(methylthio)gliotoxin (**51**), reduced gliotoxin (**52**), 6-acetyl bis(methylthio)gliotoxin (**53**), didehydrobisdethiobis (methylthio)gliotoxin (**54**) and bis-*N*-norgliovictin (**55**) (Figure 6), isolated from a culture extract of the marine-derived *N. pseudofischeri* (collection no. 2014F27-1), on HEK-29 (human embryonic kidney), HCT-116 (human colon cancer) and RKO (a poorly differentiated colon carcinoma) cell lines. However, only **46** and **49**–**52** exhibited potent cytotoxicity with IC_50_ values ranging from 0.41 to 33.56 µM, against the three cancer cell lines. The positive control, 5-fluorouracil, showed IC_50_ values of 2.04 and 45.86 µM against HCT-116 and RKO, respectively [22].

CJ 12663 (**115**) (Figure 14) and **116** (Figure 14), isolated from a culture extract of the soil-derived *N. pseudofischeri*, were assayed against KB (epidermal carcinoma of the mouth with HeLa cell contamination, ATCC CCL-17), and MCF-7 cancer cell lines. Compound **115** displayed weak cytotoxicity against KB and MCF-7 with IC_50_ values of 36.11 and 28.31 µg/mL, respectively, while **116** showed weak cytotoxicity against KB cells with an IC_50_ value of 35.23 µg/mL. Both **115** and **116** also exhibited weak cytotoxicity against Vero cells with IC_50_ values of 30.89 and 21.24 µg/mL [38].

Sartoryglabrins A (**38**), B (**39**), and C (**40**) (Figure 5), isolated from a culture extract of the soil-derived *N. glabra*, were evaluated for their capacity to inhibit the in vitro growth of MCF-7, NCI-H460 and A375-C5 cell lines using the protein binding dye SRB method. Compound **38** displayed a strong growth inhibitory activity against the MCF-7 cell line (GI_50_ = 27.0 μM) but weak inhibitory activity against the NCI-H460 cell line (G_I50_ = 84.0 μM) and inactivity against the A375-C5 cell line at the highest concentration tested (150 μM), while **39** showed moderate growth inhibitory activity against MCF-7cells, with a GI_50_ = 53.0 μM, and did not show any relevant activity (GI_50_ > 150 μM) against both NCI-H460 and A375-C5 cell lines. On the other hand, **40** exhibited moderate growth inhibitory activity, with a GI_50_ = 44.0 μM, against the MCF-7 cell line but showed weak activity against both the NCI-H460 and A375-C5 cell lines (GI_50_ = 82.3 μM and 108.0 μM, respectively). The positive control, doxorubicin, showed GI_50_ values of 42.8 nM for MCF-7; 94.0 nM for NCI-H460, and 79.5 nM for A375-C5. These results suggest that **39** is not cytotoxic since it showed selectivity toward the MCF-7 cell line [40].

Neosarphenol A (**157**) (Figure 20) and penicillide (**186**) (Figure 20), isolated from a culture extract of *N. glabra* CGMCC32286, exhibited selective and moderate cytotoxicity against the PANC-1 (human pancreatic cancer) cell line with IC_50_ values of 14.38 and 10.93 µM, respectively. The positive control, paclitaxel, showed an IC_50_ = 0.45 µM [53].

Tryptoquivaline (**56**), tryptoquivalines F (**58**), H (**59**), L (**60**), O (**61**), 3′-(4-oxoquinazolin-3-yl)spiro [1*H*]-indole-3,5′]-2,2′-dione (**67**), sartorymensin (**68**) and *epi*-fiscalin A (**70**), isolated from a culture extract of the soil-derived *N. siamensis* KUFC 6349, were evaluated for their in vitro growth inhibitory activity against Hs683, U373, A549, MCF-7, and SKMEL-28 by MTT assay. However, only **68** exhibited moderate growth inhibitory activity on the five human cancer cell lines with IC_50_ values of 50, 44, 39, 43, 73 µM, respectively. Of the positive controls, etoposide showed IC_50_ values of 4.0 µM (Hs683), 0.4 µM (U373), 4.2 µM (A549), 1.8 µM (SKMEL-28), while carboplatin showed IC_50_ values of 46 µM (Hs683), 58 µM (U373), 54 µM (A549), 69 µM (SKMEL-28) [42].

Nortryptoquivaline (**57**), tryptoquivaline F (**58**), tryptoquivaline H (**59**) (Figure 7), fiscalin A (**69**), *epi*-fiscalin A (**70**), *epi*-neofiscalin A (**72**), *epi*-fiscalin C (**74**) (Figure 8), chevalone C (**128**) (Figure 17) and 2,4-dihydroxy-3-methylacetophenone (**154**) (Figure 20), isolated from the sea-fan-derived *N. siamensis* KUFA 0017, were tested for anti-proliferative activity by MTT assay, DNA damage induction by comet assay, and the induction of cell death by nuclear condensation assay on HCT116 (colon), HepG2 (liver) and A375 (melanoma) cancer cell lines. Compounds **57**, **69**, **70**, **72**, **74**, and **128** displayed IC_50_ values in the range of 124 to 153 µM in the selected cell lines, **74** being the most active compound with IC_50_ values of 86, 24, and 75 µM for HCT116, HepG2 and A375, respectively. Doxorubicin, the positive control, showed IC_50_ values of 0.13 µM for HCT116, 0.11 µM for HepG2, and 0.08 µM for A375. Compounds **57**, **69**, and **128** also induced cell death in HCT116, while **57**, **69**, **70**, and **72** significantly induced cell death in HepG2. It was found that the induction of cell death is probably not related to genotoxicity since none of the compounds induced significant DNA damage [43].

Compounds isolated from a culture extract of the soil-derived *N. spinosa* KKU-1NK1 were also screened for cytotoxicity against KB, MCF-7, and NCI-H187 (human small lung cancer) cell lines. Tryptoquivaline L (**60**) (Figure 7), 1-hydroxychevalone C (**129**), and 1-acetoxychevalone C (**130**) (Figure 17) displayed cytotoxicity against the KB cell line with IC_50_ values of 103.3, 100.7, and 92.0 µM, respectively. Compounds **60**, **129**, **130,** and 1,11-dihydroxychevalone C (**132**) (Figure 17) displayed cytotoxicity against NCI-H187 with IC_50_ values of 42.0, 40.0, 37.2, and 39.9 µM, respectively, while tryptoquivaline (**56**) (Figure 7), **60**, **129**, **130**, and **132** showed cytotoxicity toward Vero cells with IC_50_ values of 66.5, 40.7, 39.1, 28.9, and 78.2 µM, respectively. All the tested compounds were inactive against MCF-7 cells. Doxorubicin, the positive control, showed IC_50_ values of 2.06 µM for KB, 0.16 µM for NCI-H187, and 1.39 µM for the Vero cell [44].

Brasiliamide H (**92**) (Figure 10), 7-chloro*fischeri*ndoline (**117**) (Figure 14), and aszonapyrone G (**124**) (Figure 16), isolated from a culture extract of the soil-derived *N. hiratsukae*, were assayed for their cytotoxicity against HeLa (human cervical carcinoma), KB, MCF-7, HepG2, HT-29 (colorectal adenocarcinoma) and Vero cell lines. Compound **117** exhibited weak cytotoxicity against all the tested cell lines with IC_50_ values ranging from 45 to 63 µM, while **92** and **124** were inactive. The positive control, adriamycin, showed IC_50_ values of 0.02, 2.44, 1.11, 0.37, 0.35 and 44.79 µM for HeLa, KB, MCF-7, HepG2, HT-29, and Vero cell lines, respectively [39]. Additionally, aszonapyrone A (**118**) (Figure 16), isolated from a culture extract of the soil-derived *N. tatenoi* KKU-2NK23, also exhibited cytotoxicity against NCI-H187 and KB cell lines with IC_50_ values of 4.62 and 48.18 µg/mL, respectively. Doxorubicin, the positive control, showed IC_50_ = 0.01 µg/mL against NCI-H187, and 0.33 µg/mL against KB cells [28].

### 3.2. Antibacterial and Antibiofilm Activities

Liang et al. evaluated the antibacterial activity of neosartin B (**44**), 1,2,3,4-tetrahydro-2-methyl-1,3,4-trioxopyrazino [1,2-*a*]indole (**45**), 1,2,3,4-tetrahydro-2,3-dimethyl-1,4-dioxopyrazino [1,2-*a*]indole (**46**), 1,2,3,4-tetrahydro-2-methyl-1,3,4-trioxopyrazino (1,2-*a*]indole (**47**), gliotoxin (**49**), acetylgliotoxin (**50**), bis(dethio)bis(methylthio)gliotoxin (**51**), reduced gliotoxin (**52**), 6-acetyl bis(methylthio)gliotoxin (**53**), didehydrobisdethiobis(methylthio)gliotoxin (**54**), and bis-*N*-norgliovictin (**55**) (Figure 6), isolated from a culture extract of the sea star-derived *N. pseudofischeri*, against three multidrug-resistant bacteria, i.e., the Gram-positive *Staphylococcus aureus* (ATCC 29213), the methicillin-resistant *S. aureus* (R3708), and the Gram-negative *Escherichia coli* (ATCC 25922), using a broth dilution method. However, only **49** and **52** exhibited significant inhibitory activity against these three bacteria with IC_50_ values of 12.20, 1.53, 24.53 µM, and 48.78, 1.52, 97.56 µM, respectively, against *S. aureus* (ATCC 29213), MRSA *S. aureus* (R3708), and *E. coli* (ATCC 25922). The results suggested that a disulfide bridge or reduced disulfide bond is essential for inhibitory activity, since compounds containing alkyl sulfide, such as **51**, **53**, **54**, and **55**, are void of antibacterial activity [22].

Cottoquinazolines E (**84**), F (**85**), and G (**86**) (Figure 9), isolated from a culture extract of *N. fischeri* TJ 403-CA8, were evaluated for their antibacterial activity against Gram-negative extended-spectrum β-lactamase (ESBL)-producing *E. coli*, *Acinetobacter baumannii*, *Pseudomonas aeruginosa*, *Klebsiella pneumoniae*, Gram-positive methicillin-resistant *S. aureus,* and *Enterococcus faecalis*. However, only **85** showed significant antibacterial activity against ESBL-producing *E. coli*, *A. baumannii*, *P. aeruginosa*, and *E. faecalis*, with minimum inhibitory concentration (MIC) values of 8, 32, 32, and 16 μg/mL, respectively, while **84** and **85** were inactive against all the test bacteria (MIC ≥ 100 μg/mL) [50].

Eurochevalierine (**113**), CJ-12662 (**114**), CJ-12663 (**115**) (Figure 14) and chevalone C (**128**) (Figure 17), isolated from a culture extract of the soil-derived *N. pseudofischeri*, exhibited antibacterial activity against *Bacillus cereus,* with MIC values of 64, 64, 16, and 8 µg/mL, and *S. aureus,* with MIC values of 64, 64, 128, and 16 µg/mL, respectively [38].

Aszonapyrones A (**118**) and B (**119**), sartorypyrones A (**125**) and B (**126**) (Figure 16), isolated from a culture extract of a soil-derived *N. fischeri* FO-4897, were tested for their antibacterial activity against Gram-positive and Gram-negative bacteria. Compounds **118**, **125**, and **126** displayed antibacterial activity against all tested Gram-positive bacteria viz. *B. subtilis*, *Kocuria rhizophila,* and *Mycobacterium smegmatis*, while **119** displayed antibacterial activity against only *M. smegmatis.* None of the tested compounds were active against Gram-negative bacteria, *E. coli*, and *Xanthomonas oryzae* [54].

Tryptoquivalines F (**58**), H (**59**), L (**60**), 3′-(4-oxoquinazolin-3-yl)spiro [1*H*]-indole-3,5′]-2,2′-dione (**67**) (Figure 8), and sartorypyrone C (**120**) (Figure 16) from a culture extract of the marine-sponge-associated *N. paulistensis* KUFC 7897, tryptoquivaline T (**62**) (Figure 8), aszonapyrones A (**118**) and B (**119**) (Figure 16) from a diseased coral-derived *N. laciniosa* KUFC 7896, chevalone B (**122**) (Figure 16) and chevalone C (**128**) (Figure 17) from the soil-derived *N. siamensis* KUFC 6349, sartorypyrone A (**125**) (Figure 16) from a soil-derived *N. fischeri* KUFC 6344, and sartorypyrone B (**135**) (Figure 17) from the marine sponge-associated *N. tsunodae* KUFC 9213, were evaluated for their antibacterial activity against Gram-positive *S. aureus* ATCC 25923 and *B. subtilis* ATCC 6633, and against Gram-negative *E. coli* ATCC 25922 and *Pseudomonas aeruginosa* ATCC 27853, as well as multidrug-resistant isolates from the environment. The potential synergism between these compounds and antibiotics was also evaluated against multidrug-resistant bacteria, methicillin-resistant *S. aureus* (MRSA) and vancomycin-resistant *Enterococci* (VRE). Among the meroditerpenes tested, only aszonapyrone A (**118**) and sartorypyrone A (**125**) displayed significant MIC values against Gram-positive bacteria. Compound **118** showed MIC values of 8 μg/mL against *S. aureus* ATCC 25923 and *B. subtilis* ATCC 6633, while **125** showed MIC values of 32 and 64 μg/mL, respectively, against the same reference strains. Interestingly, while **118** was active against both *S. aureus* MRSA (*S. aureus* B1 and B2) and *Enterococcus* spp. VRE isolates (*E. faecalis* W1 and W5), **125** did not show any inhibition of the growth of *Enterococcus* spp. VRE isolates in the range of concentrations tested. Very interestingly, the checkerboard method, as represented by the fractional inhibitory concentration (FIC) index, showed that a combination effect of **118** with the antibiotics oxacillin and ampicillin against MRSA and VRE isolates, respectively, was indifferent (ΣFIC > 0.5); however, **118** was able to decrease the MIC of each antibiotic tested and, thus, it may be considered as a partial synergistic effect. The association of **118** with vancomycin showed a clear synergistic effect (ΣFIC < 0.5) against the two VRE isolates (*E. faecalis* W1 and W5) tested. The combination of **125** with oxacillin and ampicillin against MRSA isolates was also found to be indifferent. Since the MIC of **125** against VRE was higher than 256 μg/mL, no checkerboard method was performed for this compound against the VRE isolates [46].

The effect of **118** and **125** at different concentrations, ranging from 2× to 1/4×MIC, on the biofilm formation by *S. aureus* ATCC 25923, *B. subtilis* ATCC 6633, and *S. aureus* B1, as well as *E. faecalis* W1 (in the case of **118**), was evaluated. All the strains tested showed no biofilm formation in the presence of 2×MIC and MIC of **118** and **125**. However, *S. aureus* ATCC 25923 and *S. aureus* B1 produced more biofilm in the presence of a sub-inhibitory concentration (1/2×MIC) of **118**. Furthermore, *S. aureus* ATCC 25923 produced a significantly higher amount of biofilm in the presence of 1/2×MIC of **118**, when compared to the control. Microscopic visualization of the biofilm produced by *S. aureus* ATCC 25923, using live/dead staining, revealed that the majority of the cells within the biofilm were viable and that large aggregates embedded in a matrix could be observed after 24 h. Interestingly, no biofilm was formed; also, no growth was observed in the presence of **118** at a concentration equal to its MIC. However, at a concentration of 1/2×MIC, it was possible to observe more biofilm in comparison to the control [46].

Examination of the structures of the meroditerpenes tested suggests the existence of some common features necessary for the antibacterial activity of this class of compounds. Although aszonapyrone A (**118**), aszonapyrone B (**119**), sartorypyrone C (**120**), chevalone B (**122**), and sartorypyrone A (**125**) all contain a 4-hydroxy-6-methyl-2*H*-pyran-2-one ring, only aszonapyrone A (**118**), chevalone B (**122**), and sartorypyrone A (**125**) have the β-acetoxyl group at C-3. In contrast to **118**, **119,** and **120**, where the 4-hydroxy-6-methyl-2*H*-pyran-2-one ring is linked to the methylene group (CH_2_-15), this ring is connected to the perhydrophenanthrene portion by an ether bridge, forming a more rigid pentacyclic structure in chevalone B (**122**). Then again, both chevalone C (**128**) and sartorypyrone B (**135**) contain a 6-methyl-4*H*-pyran-4-one ring, also connected to the perhydrophenanthrene portion by an ether bridge. Therefore, it is apparent that the presence of a free 4-hydroxy-6-methyl-2*H*-pyran-2-one ring on C-15 and the β-acetoxyl group on C-3 of the perhydrophenanthrene portion are required for the antibacterial activity of this series of meroditerpenes [46].

Harmane (**42**) (Figure 6), hopan-3β,22-diol (**96**) (Figure 12), 3β, 5α-dihydroxy (22*E*, 24*R*)-ergosta-7,22-dien-6-one (**144**) (Figure 18), chromanol derivative (**159**) (Figure 20), lumichrome (**209**) (Figure 30), isolated from the marine sponge-derived *N. tsunodae* KUFC 9213, together with dankasterone A (**148**) (Figure 19) and paecilin E (**168**) (Figure 21), which were isolated from the marine sponge-derived *N. fenelliae* KUFA0811, were tested for their antibacterial activity against Gram-positive and Gram-negative bacteria, including four reference strains, a clinical isolate sensitive to the most commonly used antibiotic families, and four multidrug-resistant isolates from the environment. Compound **168** exhibited an inhibitory effect on both *S. aureus* ATCC 29213 and *E. faecalis* ATCC 29212, with MIC values of 32 µg/mL and 16 µg/mL, respectively. However, when tested in a vancomycin-resistant (VRE) strain that was sensitive to ampicillin (*E. faecalis* A5/102), the MIC obtained was higher than that of the reference strain (64 µg/mL as opposed to 16 µg/mL). In the range of concentrations tested, **168** was ineffective against a VRE strain that was also resistant to ampicillin (*E. faecalis* B3/101). In the case of *S. aureus* strains isolated from the environment, **168** did not inhibit the growth of the bacterial strain that is sensitive to the most commonly used antibiotic families (*S. aureus* 40/61/24) as well as of MRSA *S. aureus* 66/1. However, **148** was only effective against *E. faecalis* ATCC 29212 and VRE *E. faecalis* A5/102, with MIC values of 32 µg/mL and 64 µg/mL, respectively. Compounds **148** and **168** did not exhibit any inhibitory effect on biofilm production in the four reference strains at the concentrations tested [30].

Penipurdin A (**174**) (Figure 22), tenellic acid (**182**), neospinosic acid (**183**), spinolactone (**184**), and vermixocin A (**185**) (Figure 24), isolated from the marine sponge-associated *N. spinosa* KUFA 1047, were evaluated for their antibacterial activity against reference strains (*S. aureus* ATCC 29213 and *E. faecalis* ATCC 29212) and multidrug-resistant isolates (*S. aureus* 66/1 MRSA and *E. faecalis* B3/101 VRE). However, only **184** exhibited antibacterial activity against *E. faecalis* B3/101, with a MIC value of 64 µg/mL (the positive control ceftazidime showed MIC = 8 µg/mL against *S. aureus* ATCC 29213, while kanamycin showed MIC = 32 µg/mL against *E. faecalis* ATCC 29212). Since its minimum bactericidal concentration (MBC) was more than one-fold higher than the MIC, a bacteriostatic effect was suggested for this compound. Despite not having antibacterial activity, **182** and **183** significantly inhibited biofilm formation in three of the four reference strains used in this study, viz. *E. coli* ATCC 25922 (both **182** and **183**), *S. aureus* ATCC 29213 (both **182** and **183**), and *E. faecalis* ATCC 29212 (only **182**). A more extensive effect was found for **183**, which displayed the strongest inhibitory activity on *S. aureus* ATCC 29213 [58].

Investigation of the influence of **183** in both the biofilm and its matrix spatial arrangement revealed a 98% reduction in the viability of the biofilm of *S. aureus* ATCC 29213 after 8 h of incubation with **183**. On the contrary, there was only a 10% viability reduction after 24h of incubation. An investigation of **183** on biofilm extracellular polymeric substances revealed that after 8 h of incubation, **183** increased the number of channels homogeneously distributed by the biofilm. However, after 24 h of incubation, this biofilm did not maintain its structure and appeared to be quite similar to that of the control [58].

1-Hydroxychevalone C (**129**) (Figure 17), isolated from the soil-derived *N. spinosa* KKU-1NK1, exhibited antimycobacterial activity against *Mycobacterium tuberculosis*, with a MIC value of 26.4 µM [44].

Glabramycins A (**193**), B (**194**), and C (**195**) (Figure 26), isolated from a soil-derived *N. glabra* (strains MF7030, F-155,700), were tested in the *S. aureus* antisense *rpsD* sensitized two-plate differential sensitivity assay. Compound **195** exhibited the most potent activity in this assay and showed a minimum detection concentration (MDC) of 62 µg/mL. Compound **193** was approximately four-fold less active and showed intermediate activity, with an MDC of 250 µg/mL. Compound **195** showed better activity against the panel of bacteria used in the assay, and the best activity was against *Streptococcus pneumoniae,* with a MIC value of 2 µg/mL, regardless of the medium used, while it inhibited the growth of *S. aureus* and *B. subtilis* with MIC values of 16 µg/mL but was less active against *E. faecalis* (MIC > 32 µg/mL). Compounds **193** and **194** were significantly less active than **195** [62].

Finally, 3-Methoxyglyantrypine (**2**), glyantrypine (**3**) (Figure 1), acetylaszonalenin (**9a**), 6-hydroxyacetylaszonalenin (**9b**), fischeramides A (**13**) and B (**14**) (Figure 2), tryprostatin B (**15**), 12-hydroxyfumitremorgin C (**18**), 12-methoxyfumitremorgin C (**19**), cyclotrypostatin B (**20**) (Figure 3), 6-methoxyspirotryprostatin B (**32**), spirotryprostatin C (**33**), spiro [5*H*,10*H*-dipyrrolo [1,2-*a*]:1′,2′-*d*]pyrazine-2-[3*H*], 2′[2*H*]indole]-3,5,10(1′*H*]trione (**34**) and spirotryprostatin M (**35**) (Figure 4), 7-deacetylpyripyropene A (**102**), 11-deacetylpyripyropene A **(103**) (Figure 13), and 5,6-dimethoxycircumdatin C (**211**) (Figure 30), isolated from the insect-derived *N. fischeri* TJ403-CA8, were screened for antibacterial activity against six drug-resistant microbial pathogens, including ESBL-producing *E. coli*, *A. baumannii*, *P. aeruginosa*, NDM-1-producing *K. pneumoniae*, methicillin-resistant *S. aureus* (MRSA), and *E. faecalis*. However, only **15**, **18**, **20**, **32**, **33**, **102**, and **211** displayed significant antibacterial activity against certain microbial pathogens, **211** being the most active against ESBL-producing *E. coli*, with a MIC value of 2.0 μg/mL, which was comparable to that of the clinically used antibiotic amikacin. The transmission electron microscopy (TEM) revealed that after 24 h of treatment of ESBL-producing *E. coli* with **211** at a concentration of 2 μg/mL, the cytoplasmic membranes of ESBL-producing *E. coli* cells were almost completely destroyed [20].

### 3.3. Antiviral Activity

Fischeacid (**152**) (Figure 20), fischexanthone (**175**), chrysophanol (**170)**, emodin (**171**) (Figure 22), sydowinins A (**176**) and B (**177**), AGI-B4 (**178**) (Figure 23), 3,4-dihydroxybenzoic acid (**196**) (Figure 30), adenosine (**205**), and 5′-deoxy-5′-methylaminoadenosine (**206**) (Figure 30), isolated from the marine-derived *N. fischeri* 1008F1, were tested for their effects on the replication of tobacco mosaic virus (TMV) using the leaf-disc method. The tested compounds displayed a TMV replication inhibition ranging from 36.5 to 75.9% (ribavirin, the positive control, showed 45.2% inhibition). Compounds **178** and **196** showed IC_50_ values of 0.26 and 0.63 mmol/mL, respectively [55].

*Neosartorya*dins A (**82**) and B (**83**) (Figure 8), isolated from the mangrove-derived *N. udagawae* HDN13-313, were evaluated for their activity against influenza A virus (H1N1) by cytopathic effect (CPE) inhibition assay. Compounds **82** and **83** exhibited inhibitory effects with IC_50_ values of 66 and 58 µM, respectively. The positive control, ribavirin, showed an IC_50_ = 94 µM [48].

### 3.4. Antiplasmodial Activity

Aszonapyrone A (**118**) (Figure 16) from the soil-derived *N. tatenoi* KKU-2NK23 [28], tryptoquivaline (**56**) (Figure 7), and 1-acetoxychevalone C (**130**) (Figure 17), from the soil-derived *N. spinosa* KKU-1NK1 [44], exhibited antimalarial activity against *Plasmodium falciparum* (K1, multidrug-resistant strain) with IC_50_ values of 1.34, 2.65, and 6.67 µM, respectively.

### 3.5. Anti-Inflammatory Activity

The isolated compounds from the insect-derived *N. fischeri* TJ403-CA8 were screened for their anti-inflammatory potential by observing their inhibition of nitric oxide (NO) production, induced by lipopolysaccharides (LPS) in RAW264.7 cells. However, only fischeramide A (**13**) significantly inhibited LPS-induced NO production, with an IC_50_ = 25 µM. Dexamethasone was used as a positive control [20].

### 3.6. Immunosuppressive Activity

The isolated compounds from the insect-derived *N. fischeri* TJ403-CA8 were also evaluated for their in vitro immunosuppressive activity in murine splenocytes stimulated by LPS and anti-CD3/anti-CD28 mAbs. Only fischeramide A (**13**) showed potential immunosuppressive activity in LPS and anti-CD3/anti-CD28 mAbs-activated murine splenocytes proliferation with IC_50_ values of 7.08 and 6.31 μM, respectively, while the rest of the test compounds showed no activity at concentrations up to 40 μM [20].

### 3.7. Neuroprotective Activity

Glutamate is a well-known excitable neurotransmitter that can cause neuronal cell death during acute brain insults in neurodegenerative diseases. *Fischeri*n (**153**) (Figure 20), from a culture extract of *N. fischeri* JS0553 at a concentration lower than 20 µM, was able to significantly recover the viability of mouse hippocampal neuronal (HT22) cells decreased by glutamate. Compound **153** also decreased a glutamate-induced increase in intracellular reactive oxygen species (ROS) and Ca^+^ concentration. Moreover, **153** also significantly decreased the percentage of glutamate-induced apoptotic cells, suggesting that **153** efficiently prevented glutamate-induced apoptotic HT22 cell death. Additionally, it was found that the phosphorylation of mitogen-activated protein kinases (MAPKs), i.e., ERK, JNK, and p38, as increased by glutamate, was significantly diminished by **153**, thus indicating that the inhibition of the sustained phosphorylation of MAPKs could be a key molecular mechanism of protection mediated by **153** against glutamate-induced HT22 cell death [27].

### 3.8. Lipid-Lowering Activity

Neosartoryone A (**191**) (Figure 26), isolated from a liquid culture extract of *N. udagawae* HDN13-313 by adding 5-azacytidine at 10 µM to the culture medium, was found to decrease lipid accumulation in HepG2 liver cells that was provoked by oleic acid. The effect of **191** is comparable to that of the current cholesterol-lowering drug, simvastatin, which was used as a positive control [61].

### 3.9. Enzyme Inhibitory Activities

The NADH-fumarate reductase (NFRD) system uses fumarate as a terminal electron acceptor in the mitochondrial electron transport chain and can generate ATP in the absence of oxygen. The system allows helminths to live in anaerobic circumstances inside host mammals. Since mammals do not have NFRD in their mitochondria, it is expected that a selective NFRD inhibitor could be a good anthelmintic drug candidate. Therefore, aszonapyrones A (**118**) and B (**119**), and sartorypyrones A (**125**) and D (**126**) (Figure 16), isolated from *N. fischeri* FO-5897, were tested for their inhibitory activity against mitochondrial respiratory enzymes using a submitochondrial particle of *Acaris suum* and bovine heart. Compounds **125** and **126** potently inhibited NFRD with IC_50_ values of 0.6 and 1.7 µM, respectively. They also inhibited mammalian NADH oxidase with IC_50_ values of 1.3 and 3.0 µM, respectively. Compounds **118** and **119** displayed moderate activity against NFRD with IC_50_ values of 8.7 and 72.5 µM, respectively [54].

Vermixocin A (**185**) (Figure 24), isolated from a culture extract of the marine sponge-associated *N. spinosa* KUFA 1047, exhibited anti-tyrosinase activity with a percentage inhibition of 50% at 200 µM. Since the IC_50_ value of 177 µM was obtained at lower doses (i.e., 150 and 100 µM), **185** has a moderate anti-tyrosinase activity (the positive control, galantamine, showed a percentage inhibition of 94.82% at 80 mM, and IC_50_ = 16.76 mM) [58].

### 3.10. Insecticidal Activity

PF1223 (**167**) (Figure 21), isolated from the *N. quadricincta* strain PF1223, was tested for its capacity as a non-competitive GABA receptor antagonist, which is a target for insecticide. At 2.2 µM, **164** inhibited the specific binding of [^3^H]EBOB to the housefly head membrane by 65%. It is worth mentioning that DBCPP, a non-competitive GABA receptor antagonist, displayed an IC_50_ value of 3.41 µM for the GABA housefly receptor in the [^3^H]EBOB assay [59].

Isochaetominine C (**37**) (Figure 5), 1,2,3,4-tetrahydro-6-hydroxy-2-methyl-1,3,4-trioxopyrazino [1,2-*a*]indole (**48**) (Figure 6), cadinene sesquiterpene (**98**), its deacetyl derivative (**99**), 5-formyl-6-hydroxy-8-isopropyl-2-naphthoic acid (**100**) (Figure 12), pyripyropene A (**101**), 7-deacetylpyripyropene A (**102**), 13-dehydroxypyripyropene A (**106**), phenylpyripyropenes A (**108**) and B (**109**), 5-olefin phenylpyripyropene A (**110**) (Figure 13), and 6,8-dihydroxy-3 ((1*E*,3*E*)-penta-1,3-dien-1-yl) isochroman-1-one (**165**) (Figure 21), isolated from the starfish-derived *N. pseudofischeri*, exhibited significant in vitro cytotoxicity against Sf9 cells from the insect *Spodoptera frugiperda*. Compounds **37**, **48**, **98**–**100**, **108** and **109**, at a concentration of 50 µg/L, displayed a cell growth inhibition of >90% after 48 h of treatment [21].

### 3.11. Miscellaneous

Substance P (SP) is a potent agonist and an endogenous ligand for the neurokinin-1 (NK-1) receptor subtype. It induces a variety of physiological responses, such as salivation, vasodilation, and smooth muscle contraction, and is thought to be involved in pain transmission and the inflammatory response. Therefore, selective antagonists of SP might have potential as analgesics or anti-inflammatory agents. In this context, fiscalins A (**69**) (Figure 8), B (**1**) (Figure 1), and C (**74**) (Figure 8), isolated from *N. fischeri*, were assayed for their inhibitory activity on SP. Compounds **69**, **1**, and **74** inhibited the binding of ^125^I-Bolton-Hunter SP to human astrocytoma U-373MG intact cells, with *Ki* values of 57, 174, and 68 µM, respectively [44].

The discussion of the secondary metabolites isolated from *Neosartorya* species, and their biological activities, are summarized in Table 1 and Table 2 to facilitate readers to localize the compounds of interest and to compare them between different strains of the same species or between different species. Table 1 also includes the production culture media, to allow the readers to compare not only the sources of the fungi but also the influence of the medium on the secondary metabolite profiles of the strains and species.

## 4. Conclusions and Future Perspective

The present review discusses the chemical investigation of the fungi belonging to the genus *Neosartorya*. From the literature search, 14 species (and one with no indication of a species level) have been investigated for the production of secondary metabolites. Among the most investigated species are *N. fischeri* (12 strains), followed by *N. pseudofischeri* (5 strains) and *N. glabra* (4 strains). Concerning the sources of fungi, 11 strains were isolated from the soil, 14 strains were marine-derived, one strain was insect-derived, one strain was plant-endophytic, one strain was mangrove-endophytic, and five strains were acquired from different collections. The first report of the chemical study was of *N. pseudofischeri* var. *pseudofischeri*, which was published in 1993; however, there is no indication of the source of this fungus. It is important to point out that in the early years of the chemical investigation of fungi, the identification of the fungal material was based primarily on morphological characterization. Therefore, many fungi were not identified at species level or were distinguished as different strains. It was only recently that the fungal material could also be identified by molecular techniques using internal transcribed spacer (ITS) primers. This allows taxonomists to distinguish different strains within a species level. Analysis of the literature showed an impressive chemical diversity since 213 compounds were isolated from 15 species of this genus. Moreover, the isolated compounds belong to different chemical classes and many of them possess chiral centers. The influence of the environments from which the fungi were obtained is not very clear-cut since, for the same species, marine-derived species can produce completely different metabolites from their terrestrial counterparts, as in the cases of *N. quadricincta*, *N. glabra* and *N. spinosa*, while others, such as *N. siamensis,* produced the same compounds irrespective of whether they were terrestrial or marine-derived. Besides the source of the fungi, the production culture medium can also play an important role in secondary metabolites production, as can be seen by the incorporation of dimethyl sulfoxide in the secondary metabolites by *N. udagawae* HDN13-313.

Interestingly, Voser et al., in their recent review using fingerprint cluster analysis, based on the MarineLit database, which covers compounds isolated from the marine environment between 1956 and 2020, and the NPAtlas database, which contains compounds isolated from microorganisms and published between 1877 and 2020, have found that marine fungal natural products (NPs) are nested with terrestrial fungal NPs at a relatively high proportion (74.6%). This indicates that marine and terrestrial fungi are more likely to share common biosynthetic gene clusters, or that marine sources of fungi are likely to be terrestrial “wash-ins” [65]. The authors have also found that most studies used potato dextrose or rice in sea water or sea salt as culture media for most of the marine-derived fungi. This is also true in the case of the culture media used to culture *Neosartorya* species, as shown in Table 1. Therefore, the production of unique and different NPs by terrestrial and marine-derived fungi in the future must rely on new culture techniques and new technologies such as genome mining.

## Figures and Tables

**Table 1:** *Neosartorya* species (and strains), sources, production medium, isolated compounds, and references.

*Neosartorya* Species and Strains	Sources	Culture Medium	Isolated Compounds	References
*N. fischeri* var. *fischeri* CBM-FA-0156	No source	Solid sterile rice	**24, 26, 153**	[34]
*N. fischeri*	Plant rhizosphere	Liquid medium (glycerol, dextrin, Bacto-soytone, yeast extract)	**1, 69, 73**	[18]
*N. fischeri*	River sediment	Liquid medium (glycerol, glucose, corn steep liquor)	**179**	[60]
*N. fischeri* IFM 52672	No source	Solid medium containing moist rice	**8, 57, 150, 151**	[55]
*N. fischeri* KUFC 6344	Coastal forest soil	Solid cooked rice	**8, 9a, 11, 25, 97, 118, 125**	[25]
*N. fischeri* CGMCC 3.5378	Chinese Academy of Science	Solid medium containing dried wheat bran	**16, 17, 20, 21, 22, 27, 28, 29, 30**	[32]
*N. fischeri* CGMCC 3.5378	Chinese Academy of Science	Solid medium containing dried wheat bran	**16, 31,139, 140**	[31]
*N. fischeri* CGMCC 3.5378	Chinese Academy of Science	Solid medium containing moist corn germ	**8, 9, 10, 24, 27**	[26]
*N. fischeri* FO-5897	Soil sample	Solid sodden rice	**118, 119, 125, 126**	[54]
*N. fischeri* NRRL 181	DSMZ (DE-Braunschweig)	Moist wheat bran	**84, 85, 101**	[49]
*N. fischeri* NRRL 181	DSMZ (DE-Braunschweig)	Liquid medium containing potato dextrose agar	**101, 102, 104, 105, 139, 142, 143, 148, 149,**	[33]
*N. fischeri* JS0553	Endophytic fungus isolated from the plant *Glehnia littoralis* (Family Apiaceae)	Solid rice medium	**8, 9a, 20, 26, 101, 125, 126, 127, 153**	[27]
*N. fischeri* TJ 403-CA8	The insect *Cryptotympana atrata*	Solid rice medium	**84, 85, 86**	[50]
*N. fischeri* TJ 403-CA8	The insect *Cryptotympana atrata*	Solid rice medium	**2, 3, 9a, 9b, 13, 14, 18, 19, 102, 211**	[20]
*N. fischeri* 1008F1	Marine-derived	Solid rice, glucose-peptone-yeast medium	**152, 170, 171, 175, 176, 177,178, 205, 206**	[55]
*N. fischeri*	Marine mud	Liquid medium containing barley sugar, glucose, yeast extract, mannitol, ajinomoto	**1,78, 79**	[19]
*N. pseudofischeri* KUFC 6422	Soil sample	Solid sterile rice	**36, 89, 90a/90b, 98, 103, 113, 189**	[35]
*N. pseudofischeri* strain CBS 404.67	Centraal Bureauvoor Schimmelcultures of Baan	Solid medium (rice) and liquid medium (M1-D)	**49, 51, 98, 101, 107, 113, 117**	[41]
*N. pseudofischeri* (collection 2014F27-1)	Inner tissue of sea star (*Acanthaster planci*)	Liquid media: glycerol-yeast extract (GlyPY) and glycerol-yeast extract (GluPY)	**6, 43, 47, 51, 53, 54**	[22]
*N. pseudofischeri*	Inner tissue of starfish (*A. planci*)	Liquid medium (glucose-peptone, yeast extract)	**4, 5, 37, 41, 48, 98–102, 106, 108–110, 165, 169**	[21]
*N. pseudofischeri*	Soil sample	Potato dextrose liquid medium	**37, 64, 90a/b, 91, 101, 113–116, 121, 122, 128, 131, 133, 134, 136**	[38]
*N. glabra*	Soil sample	WS80 (whole wheat flour, xylose, fructose)	**190, 191, 192**	[62]
*N. glabra*	Soil sample	Cooked rice solid medium	**38, 39,49**	[40]
*N. glabra* CGMCC32286	Chinese Academy of Science	Dried wheat bran	**111, 112, 157–160, 166. 186–188**	[53]
*N. glabra* KUFA0702	Marine sponge *Mycale* sp.	Cooked rice solid medium	**7, 8,12, 87, 88, 93–95**	[23]
*N. tsunodae* KUFC 9213	Marine sponge *Aka coralliphaga*	Cooked rice solid medium	**97, 125**	[25]
*N. tsunodae* KUFC 9213	Marine sponge *Aka coralliphaga*	Cooked rice solid medium	**42, 96, 97, 125, 128, 144, 162, 207, 209**	[30]
*N. laciniosa* KUFC 7896	Diseased coral *Porites lutea*	Cooked rice solid medium	**60, 62, 67, 118, 119**	[25,46]
*N. paulistensis* KUFC 7897	Marine sponge *Chondrilla austroliensis*	Cooked rice solid medium	**58–60, 67, 120, 210**	[46]
*N. fenelliae* KUFA 70811	Marine sponge *Cathria reinwardtii*	Cooked rice solid medium	**8, 87, 97, 122, 145–148, 168, 180, 207, 208**	[30]
*N. siamensis* KUFC 6364	Soil sample	Cooked rice solid medium	**56, 58–61, 67, 68, 69–74, 154**	[42]
*N. siamensis* KUFA 0017	*Sea fan (Rumphella* sp.)	Cooked rice solid medium	**57, 58, 59, 69, 70, 72, 74, 128, 154**	[43]
*N. takakii* KUFC 7898	Marine alga *Amphiroa* sp.	Cooked rice solid medium	**8, 9a, 12, 58, 59, 60, 63, 67, 137, 163**	[29]
*N. spinosa* KKU-1NK1	Soil sample	Malt extract peptone broth	**56, 57, 60, 77, 122, 128–133, 155, 156**	[44]
*N. spinosa* KUFA 1047	Marine sponge *Mycale* sp.	Cooked rice solid medium	**163, 172, 173, 174, 182–185**	[48]
*N. quadricincta* strain 1PF1223	The Meiji Seika Kaisha Collection	Water-soaked raw rice and soybean meal	**167**	[59]
*N. quadricincta* KUFA 0081	Marine sponge *Clathria reinwardti*	Cooked rice solid medium	**161, 164, 197–203**	[57]
*N. udagawae* HDN13-313	Mangrove plant *Aricennia marina*	Liquid medium (maltose, mannitol, glucose, monosodium glutamate)	**73, 80, 81, 82, 83**	[48]
*N. udagawae* HDN13-313	Mangrove plant *Aricennia marina*	Liquid medium (glucose, peptone +5-azacytidine)	**190–192**	[48]
*N. hiratsukae*	Soil sample	Liquid medium (potato dextrose)	**37, 90a/90b, 92, 101, 113–115, 117, 121–128, 133, 181, 189, 212**	[39]
*N. tatenoi* KKU-2NK23	Soil sample	Liquid medium (Potato dextrose)	**8, 118, 119, 138, 139, 213**	[28]
*Neosartorya* sp. HN-M-3	Marine mud	Liquid medium (barley sugar, glucose, yeast extract	**65, 66**	[47]

**Table 2:** Biological activity of the isolated compounds from *Neosartorya* species.

Activity	Type of Cells/Organisms	Compounds	Reference
Anticancer/cytotoxic activity	MCF-7, NCI-H460, A375-C5 SGC-7901, BEL 7404 HL-60 Hs683, U373, A549, MCF-7, OE21, SKMEL28 Hs683, U373, A549, MCF-7, B16F10, SKMEL28 Hs683, U373, A549, MCF-7, SKMEL-28 HEK-29, HCT-116, RKO KB, MCF-7, Vero cells MCF-7, NCI-H460, A375-C5 PANC-1 HCT116, HepG2, A375 KB, MCF-7, NCI-H187 HeLa, KB, MCF-7, HepG2, HT-29,Vero cell NCI-H187, KB	**118, 119, 125, 135**	[25]
		**178**	[55]
		**3, 78, 79**	[19]
		**98, 113**	[35]
		**49, 107, 116**	[41]
		**68**	[42]
		**46, 49–52**	[22]
		**115, 116 (weak)**	[38]
		**38** (MCF-7, NCI-H460), **39** (MCF-7), **40** (MCF-7) **157,186**	[40]
			[53]
		**57, 69, 70, 72, 74, 128**	[43]
		**60, 129, 130, 132**	[44]
		**117**	[39]
		**118**	[28]
Antibacterial activity	*S. aureus* ATCC 29213, *S. aureus* MRSA, *E. coli* ATCC 25922 *E. coli* ESBL, *A. baumannii*, *P. aeruginosa*, *E. faecalis* *S. aureus*, *B. cereus* *B. subtilis*, *Kocuria rhizophila*, *Mycobacterium smegmatis*, *S. aureus* ATCC 25923, *B. subtilis* ATCC 6633, *S. aureus* MRSA, *E. faecalis* VRE *S. aureus* ATCC 29213, *E. faecalis* ATCC 29212, *S. aureus* ATCC 29213, *E. faecalis* ATCC 29212, *S. aureus* MRSA, *E. faecalis* VRE *Streptococcus pneumoniae*, *S. aureus*, *B. subtilis*, *E. faecalis* *E. coli* ESBL, *A. baumannii*, *P. aeruginosa*, NDM-1-producing *K. pneumoniae*, *S. aureus* MRSA, *E. faecalis*	**49, 52**	[22]
		**85**	[50]
		**113, 114, 115, 128**	[38]
		**118, 119** (*M*. *smegmatis*), **125, 126**	[54]
		**118, 125**	[46]
		**168**	[30]
		**184**	[58]
		**193, 194, 95**	[62]
		**15, 18, 20, 32, 33, 102, 211**	[20]
Antibiofilm activity	Inhibition of biofilm formation in *S. aureus* ATCC 25923, *B. subtilis* ATCC 6633 and *S. aureus* B1, *E. faecalis* W1 Inhibition of biofilm formation in *E. coli* ATCC 25922, *S. aureus* ATCC 29213, *E. faecalis* ATCC 29212 (only 182)	**118, 125**	[46]
		**182, 183**	[58]
Antiviral activity	Inhibition of replication of tobacco mosaic virus (TMV) Inhibitory effects against influenza A virus (H1N1)	**178, 196**	[55]
		**82, 83**	[48]
Antiplasmodial activity	Antimalarial activity against *Plasmodium falciparum* (K1, multidrug-resistant strain)	**56, 118, 130**	[28,44]
Anti-inflammatory activity	Inhibition of nitric oxide (NO) production induced by lipopolysaccharide (LPS) in RAW264.7 cells	**13**	[20]
Immunosuppressive activity	Immunosuppressive activity in LPS and anti-CD3/anti-CD28 mAbs-activated murine splenocytes	**13**	[20]
Neuroprotective activity	Decrease a glutamate-induced increase in intracellular reactive oxygen species (ROS) and Ca^+^ concentration and prevention of glutamate-induced apoptotic HT22 cell death	**153**	[27]
Lipid-lowering activity	Decrease the lipid accumulation in HepG2 liver cells triggered by oleic acid	**191**	[61]
Enzyme inhibitory activity	Inhibition of NADH-fumarate reductase (NFRD) In vitro anti-tyrosinase activity	**118, 119, 125, 126**	[54]
		**185**	[58]
Insecticidal activity	Inhibition of the specific binding of [^3^H]EBOB to housefly head membrane In vitro cytotoxicity against Sf9 cells from the insect *Spodoptera frugiperda*	**167**	[59]
		**98–100, 108, 109**	[21]
Miscellaneous	Inhibition of the binding of ^125^I-Bolton-Hunter substance P to human astrocytoma U-373MG intact cells	**1, 69, 74**	[44]

## Data Availability

Data sharing is not applicable to this article.
